# Identification of Potent Chemotypes Targeting *Leishmania major* Using a High-Throughput, Low-Stringency, Computationally Enhanced, Small Molecule Screen

**DOI:** 10.1371/journal.pntd.0000540

**Published:** 2009-11-03

**Authors:** Elizabeth R. Sharlow, David Close, Tongying Shun, Stephanie Leimgruber, Robyn Reed, Gabriela Mustata, Peter Wipf, Jacob Johnson, Michael O'Neil, Max Grögl, Alan J. Magill, John S. Lazo

**Affiliations:** 1 University of Pittsburgh Drug Discovery Institute and the Pittsburgh Molecular Library Screening Center, Pittsburgh, Pennsylvania, United States of America; 2 Departments of Pharmacology and Chemical Biology, University of Pittsburgh, Pittsburgh, Pennsylvania, United States of America; 3 Department of Computational Biology, University of Pittsburgh, Pittsburgh, Pennsylvania, United States of America; 4 Department of Chemistry, University of Pittsburgh, Pittsburgh, Pennsylvania, United States of America; 5 Walter Reed Army Institute of Research, Silver Spring, Maryland, United States of America; University of Pittsburgh, United States of America

## Abstract

Patients with clinical manifestations of leishmaniasis, including cutaneous leishmaniasis, have limited treatment options, and existing therapies frequently have significant untoward liabilities. Rapid expansion in the diversity of available cutaneous leishmanicidal chemotypes is the initial step in finding alternative efficacious treatments. To this end, we combined a low-stringency *Leishmania major* promastigote growth inhibition assay with a structural computational filtering algorithm. After a rigorous assay validation process, we interrogated ∼200,000 unique compounds for *L. major* promastigote growth inhibition. Using iterative computational filtering of the compounds exhibiting >50% inhibition, we identified 553 structural clusters and 640 compound singletons. Secondary confirmation assays yielded 93 compounds with EC_50_s ≤ 1* µ*M, with none of the identified chemotypes being structurally similar to known leishmanicidals and most having favorable *in silico* predicted bioavailability characteristics. The leishmanicidal activity of a representative subset of 15 chemotypes was confirmed in two independent assay formats, and *L. major* parasite specificity was demonstrated by assaying against a panel of human cell lines. Thirteen chemotypes inhibited the growth of a *L. major* axenic amastigote-like population. Murine *in vivo* efficacy studies using one of the new chemotypes document inhibition of footpad lesion development. These results authenticate that low stringency, large-scale compound screening combined with computational structure filtering can rapidly expand the chemotypes targeting *in vitro* and *in vivo Leishmania* growth and viability.

## Author Summary

Leishmaniasis is a parasitic disease with cutaneous, mucocutaneous and visceral clinical manifestations, depending on the *Leishmania spp.* and human host. Globally, there are 350 million people at risk of leishmaniasis, but current treatment options rely predominantly on ancient pentavalent antimonials, which have the potential to cause serious systemic toxicity. Our research focuses on the rapid expansion of potential anti-leishmanial compounds that could function as novel chemical structures for future drug development and offer additional therapeutic options to patients with leishmaniasis. We combined high throughput screening methodologies with computational algorithms and multiple confirmatory assay formats to identify and characterize new potent *L. major* promastigote growth inhibitors, including one that displays *in vivo* activity without toxicity to human cells. Our use of a large, broadly distributed compound library enabled the identification of these new chemotypes. In addition, since this chemical library is publicly available and annotated, we were able to cross-query archived bioassays and to identify new molecular targets that may be involved in *L. major* growth and viability as well as identify new protein targets for future leishmanicidal drug discovery.

## Introduction

Leishmaniasis is endemic in >85 developing countries with >1.5 million estimated cases occurring each year and an additional 350 million people at risk of infection [1]. Increased travel and migration within the tropics, subtropics, Middle East and Southern Europe as well as global climate and environmental changes are making leishmaniasis a considerable risk for populations in geographic regions previously unaffected by the disease [2]–[5]. As a result, there has been a progressive expansion of leishmaniasis endemic regions as well as a concomitant increase in the total number of reported leishmaniasis cases, often in epidemic proportions (*i.e.*, with 100,000–200,000 individuals infected) [6]–[9]. Transmission of leishmaniasis most commonly occurs via an infected phebotomine sandfly. Leishmaniasis can also be transmitted, albeit rarely, through blood transfusions, especially to individuals with immature or compromised immune systems, further expanding and globalizing the number of at-risk populations [10]. With clinical manifestations ranging from cutaneous (CL) and mucocutaneous (M-CL) to visceral, leishmaniasis has profound cultural and socioeconomic repercussions due to overt disability, disfigurement or scarring, and death [4], [11]–[15].

Despite the prevalence of leishmaniasis and its impact on human life, there are no vaccines or prophylactic drugs for any form of the disease. Current chemotherapeutic treatments rely heavily on the use of the pentavalent antimonials, sodium stibogluconate, and meglumine antimoniate, which were first introduced more than a half century ago [16]–[18]. Significantly, these compounds have been used without refinement for decades, have serious side effects and are declining in efficacy due to chemoresistance [19]–[22]. Second-line drugs, such as pentamidine and amphotericin B, are available but they too have significant untoward effects and pharmacological liabilities [4],[18]. Moreover, these existing leishmanicidals often require continuous clinical surveillance, have invasive or painful routes of administration and, are expensive for endemic areas. Others have attempted to augment the pool of available leishmanicidals by exploiting drugs approved for other diseases. Among newer treatments are the use of rifampicin, tamoxifen, doxocycline, monomycine, trimethoprim and nifurtimox; however, these agents are generally associated with limited anti-leishmanial efficacy [18], [23]–[29]. To maximize effectiveness and minimize toxicity, the choice of drug dosage and duration of therapy should be individualized based on the region of disease acquisition and host factors such as immune status. Also, we know that some drugs and regimens are effective only against certain *Leishmania* species or strains and only in certain areas of the world. The idea that one drug might treat all forms of leishmaniasis has rapidly lost popularity. Regrettably, there is a paucity of large-scale drug discovery efforts focusing on the design of new small molecules (*i.e.* drugs) that can treat individuals with leishmaniasis. This deficiency has contributed to leishmaniasis being classified as a neglected disease, with CL being the most neglected among the clinical manifestations of leishmaniasis [13]. Thus, there is a strong need to identify potential new drug treatments for specific clinical manifestations of leishmaniasis, and especially novel chemotherapeutics for CL.

As with other pathogenic diseases, genetic tools and genomic sequencing information are now available for multiple *Leishmania spp.* enabling a molecular target- driven approach to anti-leishmanial drug discovery [30]–[32]. Nonetheless, the low success rate of those efforts may reflect an incomplete understanding of the complexities of leishmaniasis and the significance of the proposed molecular targets to parasite growth or survival [33],[34]. Thus, whole parasite phenotypic anti-leishmanial drug discovery remains appealing. Until recently, however, most efforts to identify new leishmanicidals via whole parasite screening have concentrated on the exploitation of limited, small-scale activities using discrete, focused compound sets or compounds with known pharmacological actions [35],[36]. Consequently, the identification of novel leishmanicidal chemotypes has been effectively limited by screening throughput as well as compound library diversity. We postulate that the identification of new anti-leishmanial chemotypes can be rapidly accelerated by using low stringency, high throughput screening (HTS) methodologies with large diverse compound libraries combined with computational tools. For maximum utility, the HTS assays should be well-validated, integrated with data management and capture systems, have a simple assay format, be relatively inexpensive and, be coupled with secondary assays to expedite confirmation of the activity and specificity of novel chemotypes [37]–[39].

In the work presented herein, we developed and implemented a multi-tiered compound screening paradigm to identify and confirm novel leishmanicidal chemotypes. Our screening strategy was founded on a validated *L. major* (taxonomy id 5664) promastigote drug susceptibility HTS assay, which we used to screen a structurally diverse 196,146 compound library at low stringency (*i.e.*, a relatively high compound screening concentration - 10 µM). Promastigotes are easy to use and there is evidence that they provide a good model for gauging a compound's leishmanicidal activity [40]–[42]. The selected assay detection reagent, alamar blue, is simple, inexpensive, easily adapted to automated HTS procedures and has been frequently used to identify and characterize leishmanicidal compounds [43],[44]. Our primary aim was to maximize the potential chemical diversity of the *L. major* promastigote growth inhibitory chemotypes identified. Thus, we purposefully screened a large chemical library at a relatively high initial compound concentration to yield the maximum number of active compounds. To reduce the candidate compounds to a manageable size, we exploited computational methods to cluster chemotypes. We termed this integrated approach HILCES for high throughput, low-stringency, computationally enhanced small molecule screening. Representative members of each cluster and the unassigned compounds, *i.e.* singletons, were then sequentially characterized with respect to potency, specificity of response, and predicted *in silico* ADMET. Significantly, the use of an annotated public compound library enabled us to determine compound specificity by comparing its bioactivity in up to 369 additional biochemical or phenotypic assays. Moreover, specific molecular targets were suggested that might be critical to *Leishmania* growth, viability and survival. Selected compounds also demonstrated *in vivo* efficacy in a murine model system.

## Materials and Methods

### Chemicals and reagents

Black, clear bottom tissue culture treated 384-well microtiter plates were purchased from Greiner (Monroe, NC) and used for all experiments. Alamar blue (Cell Titer Blue) was purchased from Promega (Madison, WI); tamoxifen from MP Biomedicals (Solon, OH); dimethyl sulfoxide (DMSO), aphidicolin from Sigma-Aldrich (St. Louis, MO); phenyltoloxamine, clotrimazole, sangivamycin and amphotericin B from VWR (West Chester, PA); disulfiram from Fisher Scientific (Pittsburgh, PA); pentamidine from Toronto Research Chemicals (Ontario, Canada) and; acivicin from Biomol (Plymouth Meeting, PA). The PubChem CID compounds 786799, 742546, 760847, 2946668, 757789, 2851545, 728862, and 16187595 were obtained from Chembridge (San Diego, CA). All purchased compounds were subjected to quality control testing by their respective manufacturers.

### Routine *L. major* parasite culturing and counting

*L. major* promastigotes (MHOM/SA/85/JISH118) (a kind gift from Dr. Frederick Buckner) were maintained in Medium 199 (pH 7.2) (Invitrogen, Carlsbad, CA) supplemented with 10% heat-inactivated fetal bovine serum (FBS) (Hyclone, Logan, UT), penicillin (100 units/mL) and streptomycin (100* µ*g/mL) as previously described in Buckner and Wilson [45]. Promastigotes were grown in vented T75 tissue culture flasks and maintained at 28°C. Promastigote cultures were initiated at 10^5^ parasites per mL and subcultured every 3–4 days. *L. major* promastigote counts were performed in duplicate using a hemocytometer and particle counter (Beckman Coulter, Fullerton, CA). For HTS assays, *L. major* promastigote cultures were harvested during exponential growth phase (∼2.0–3.0×10^7^ parasites/mL) and were not maintained past passage 20.

Axenic amastigote-like parasite populations were derived from stationary growth phase *L. major* promastigotes and were maintained in Schneider's medium (pH 4.9) supplemented with 10% heat-inactivated FBS, penicillin (100 units/mL), streptomycin (100 µg/mL), L-glutamine (2 mM) and cultured at 32°C with 5% CO_2_. This parasite population was specifically designed to test the potency of compounds under low pH conditions. At these culturing conditions ∼80–90% of the *L. major* parasites exhibited an aflagellated rounded morphology and displayed similar characteristics of previously described axenic amastigotes including, but not limited to doubling time (*i.e.*, ∼24 h), clustered growth patterns, agglutination response to PNA lectin, protease activity and protein expression profiles [46]–[49]. Characterization of this parasite population also includes genotyping studies to confirm identity. All axenic amastigote-like parasite cultures were maintained in vented T25 or T75 flasks. For drug susceptibility assays, axenic amastigote-like parasites were harvested in exponential growth phase.

### Compound libraries

The library of pharmacologically active compounds (LOPAC) (1,280 compounds) was purchased from Sigma-Aldrich. The DP validation set (159 compounds) and the University of Pittsburgh Chemical Methodology and Library Development Center (UP-CMLD) diversity set (960 compounds) were obtained from the UP-CMLD (http://ccc.chem.pitt.edu/UPCMLD/index.html). We assayed the 196,146 compound library from the Pittsburgh Molecular Libraries Screening Center (PMLSC) for *L. major* growth inhibitors. Cherry-picked compounds from the PMLSC library were supplied by BiofocusDPI (San Francisco, CA).

### Library compound dilution scheme for primary screening

In primary screening, 2* µ*L of a 1 mM test compound solution in 100% DMSO were diluted in 22* µ*L complete *L. major* promastigote growth medium, generating an 83.3* µ*M working concentration (in 8.3% DMSO) of library compounds. The final test compound concentration was 10* µ*M with a constant DMSO concentration of 1% in each assay well.

### Automated primary HTS using *L. major* drug susceptibility assay

The *L. major* promastigote drug susceptibility assay was performed in a final volume of 25* µ*L using our previously described 384-well microtiter plate format [38],[39]. For automated HTS procedures, *L. major* promastigotes (5,000 parasites/22* µ*L) in complete growth medium were seeded into each well of the microtiter plates using a MAPC2 bulk dispenser (Titertek, Huntsville, AL). Test and control compounds (3* µ*L) were added to individual wells using a Velocity 11 V-prep (Menlo Park, CA) liquid handling system, equipped with a 384-well dispensing head, followed by centrifugation at 50 *g* for 1 min. Negative (vehicle) controls contained 1% DMSO, positive controls contained 10% DMSO and EC_50_ controls contained 500 nM tamoxifen (final well concentrations). Assay plates were allowed to incubate for 44 h at 28°C in the presence of 5% CO_2_. Five *µ*L of alamar blue reagent were added to each assay plate well and incubated for 4 h at 28°C with 5% CO_2_. Data were captured on a Molecular Devices SpectraMax M5 (excitation_560_; emission_590_). Individual assay plate Z-factors were derived from the vehicle and positive controls, and data from plates were used only if Z-factors were >0.5 [50]. Primary hits were defined as compounds displaying ≥50% inhibition of signal readout. The *L. major* axenic amastigote-like assay was performed using the alamar-blue assay format and detection methods as the promastigote except that assay plates (7,500 parasites/well) were incubated for 144 h at 32°C in the presence of 5% CO_2_.

### Potency determinations

In initial 10-point EC_50_ determination experiments, two *µ*L of 1 mM test compound in 100% DMSO were diluted with 46* µ*L complete *L. major* promastigote growth medium creating a 41.7* µ*M working concentration of library compounds. A two-fold serial dilution was then performed creating a concentration range (0.08–41.7* µ*M). The assays were performed in duplicate with a final 10-point concentration range spanning 0.01–5.00* µ*M. A compound was designated a confirmed inhibitor only if the EC_50_ values of both replicates were ≤5* µ*M.

### Flow cytometer-based growth inhibition and cytotoxicity assays

*L. major* promastigotes were harvested in exponential growth phase and adjusted to a concentration of 2.1×10^5^ parasites per mL in complete growth medium. Fifteen thousand parasites (75* µ*L volume) were then seeded into each well of a 96 well microtiter plate and were treated with a concentration range (0.1–50* µ*M) of test and control compounds. Parasite assay plates were incubated for 48 h at 28°C. Samples were prepared by transferring five *µ*L of parasite suspension to 100* µ*L of ViaCount reagent (Guava Technologies, Hayward, CA) followed by gentle and thorough mixing to ensure an even distribution of parasites. Data were captured on a Guava EasyCyte Plus flow cytometer and analyzed using CytoSoft 5.0.2 software (Guava Technologies) and GraphPad Prism 5.0 software (San Diego, CA). A total of 500–1,500 parasites were evaluated in duplicate per compound treatment.

### Mammalian cell line-based specificity assays

Mammalian cells were cultured and maintained according to ATCC specifications (ATCC, Manassas, VA). Cell line drug susceptibility assays were performed in final volumes of 25* µ*L using our previously described 384-well microtiter plate format [38],[39]. Briefly, for automated HTS procedures, cells (A549, IMR-90 and, HeLa, 1,000 cells; PC-3, 750 cells and; MDA-MB-231, 3,000 cells) in complete culture medium were seeded into each well of 384-well microtiter plates using a Titertek MAPC-2 bulk dispenser. Test and control compounds were added to individual wells as described above. Vehicle and positive controls were 1% DMSO and 10% DMSO, respectively (final well concentrations). Assay plates were incubated for 44–46 h at 37°C in the presence of 5% CO_2_ and growth inhibitory effects were determined as described above. Five *µ*L of alamar blue reagent was added to each well and incubated for 2–4 h. Data were captured as described above.

### HTS data analysis, computational filtering, and statistical analysis

Primary HTS data analysis and subsequent compound EC_50_ calculations were performed using ActivityBase (IDBS, Guilford, UK) and Cytominer (University of Pittsburgh Drug Discovery Institute, Pittsburgh, PA). To maximize the diversity of leishmanicidals, we performed the primary HTS assay at low stringency with 10* µ*M of each compound, which ensured a high rate of positive compound identification. Jarvis-Patrick clustering methodology (Leadscope, Columbus, OH) was used to computationally filter the number of compounds that proceeded through secondary hit confirmation assays [51]. This deterministic and non-iterative methodology generated non-overlapping, non-hierarchical clusters based on chemical structural similarities. The algorithm selected the number of clusters, with each cluster consisting of at least one structure, and generated non-overlapping, non-hierarchical clusters. A compound with the smallest maximum pairwise distance to the other cluster members was selected as the representative for the structural cluster. In clusters with only two compounds, either compound was selected to represent its specific cluster. This methodology enabled us to reduce the number of potential inhibitors to be evaluated from ∼20,000 to ∼1,200 (0.61% hit rate) while maximizing the chemical diversity of the primary hit pool. Additional data visualization and statistical analysis were performed using Graphpad Prism software 5.0 and Spotfire (Somerville, MA). The PubChem database (http://PubChem.ncbi.nim.nih.gov) was mined to determine if the confirmed *L. major* growth inhibitors exhibited bioactivity in other assays. In some instances, select compounds were tested in approximately 300 additional assays, including various molecular target based, phenotypic and cytotoxicity assays. The structural similarity of the confirmed inhibitors was determined using Leadscope software (*i.e.* Tanimoto score).

### Predicted drug-like properties of confirmed *L. major* growth inhibitors

Confirmed *L. major* growth inhibitors were filtered further for desirable drug-like properties using ADME Boxes v4.0 software (Pharma Algorithms, Toronto, Canada) [52],[53]. In brief, this algorithm predicted human adsorption and metabolism bioavailability for new compounds using a combination of two methods: probabilistic and mechanistic. A bioavailable compound was defined as one that should satisfy the following criteria: dissolve in the stomach or intestine under variable pH, withstand acid hydrolysis at pH<2, permeate through intestinal membrane by passive or active transport, withstand P-glycoprotein efflux in concert with metabolic enzymes in intestine, and withstand first-pass metabolism in liver. Based on predictions, oral bioavailability was classified as follows: poor<30%; moderate 30–70%; and good >70%. The ADME Boxes software also was used to predict toxicity (*i.e.* AMES, hERG, skin irritation, LD_50_ in mice and Cyp450 inhibition) of compounds. For genotoxicity, we calculated the probability that a compound would register as a positive in an Ames mutagenicity screening test while hERG *in silico* assessment was calculated as the probability of a compound being a hERG channel inhibitor at clinically relevant concentrations. Acute toxicity was estimated as the LD_50_ value (mg/kg) after intraperitoneal, oral, intravenous or subcutaneous administration to mice. Skin irritation *in silico* predictions reflected measurements usually performed in a rabbit Draize test, which primarily measures the toxicity of a compound intended for topical application, cosmetic use or possibly coming into contact with human skin at a standard dose (100 or 500 mg). Toxicity predictions have an associated Reliability Index (RI) as defined as follows: RI<0.3; not reliable, RI = 0.3–0.5, borderline reliability; RI = 0.5–0.75, moderate reliability and RI≥0.75, high reliability [53].

### *In vivo* murine CL efficacy studies

Adult female Balb/c mice (6 to 10 week old) were obtained from (Charles River Laboratories, Wilmington, MA) and maintained as outlined by the National Institutes of Health Guide for the Care and Use of Laboratory Animals. All *in vivo* studies were carried out in accordance with protocols approved by the Institutional Animal Care and Use Committee (IACUC) at the University of Miami (IACUC number C01-08). Food and water were supplied *ad libitum*. Mice were anesthetized prior to subcutaneous inoculation with 10^6^ stationary phase *L. major* parasites in 50* µ*L of Dulbecco's modified Eagle medium in the left hind footpad. Animals were examined daily to determine lesion development. Mice were treated with experimental compounds at a concentration of 40 or 160 mg/kg in a 200-*µ*L total volume/mouse. Control mice were injected with an equivalent amount of vehicle control or amphotericin B (12.5 mg/kg). Footpad lesion size was measured using a Vernier caliper at 7, 14, and 21 days post-compound administration. Mice were euthanized in a CO_2_ chamber at day 21.

## Results

### HTS assay optimization procedures and validation of the low stringency screening strategy

**Figure 1:**
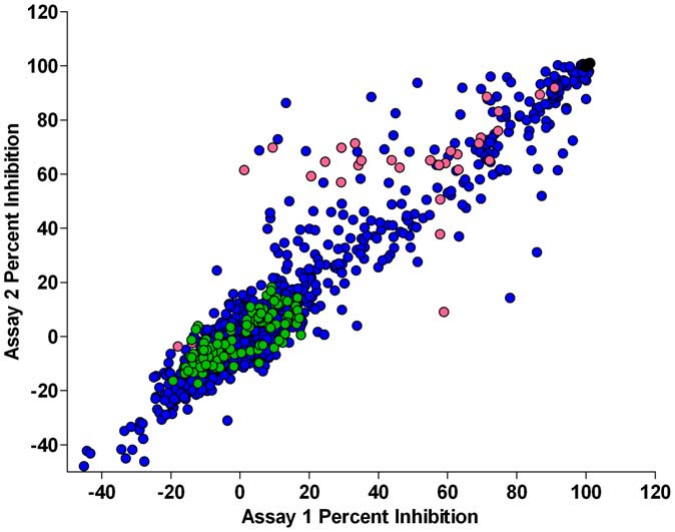
Reproducibility of the automated assay format demonstrated with the Library of Pharmacologically Active Compounds (LOPAC). The robustness of the *L. major* promastigote drug susceptibility assay was demonstrated by screening the 1,280 compound LOPAC library in duplicate at 10* µ*M. The reproducibility between the two assays was R^2^ = 0.94. Average Z-factors equaled 0.71±0.03 with a signal to background (S∶B) ratio of 20.98±0.32. (blue circle - test compound; green circle - MAX control; red circle - MIN control; and pink circle - EC_50_ control).

The growth characteristics of the *L. major* promastigotes in a 384-well plate format were first optimized. When promastigotes were seeded at 10^5^/mL on day 0, the parasite exhibited conventional exponential, stationary and declining phases over seven days, as anticipated from previous reports with other plate formats [54] (Figure S1). All subsequent assay development and screening studies were performed with exponentially growing *L. major* promastigote cultures (∼2–3×10^7^ promastigotes/mL). Promastigotes readily tolerated up to 1% DMSO with no degradation of growth rate, and the optimal incubation time for alamar blue was 4 h. In the 384-well format, the EC_50_ for amphotericin B was 207±11 nM, consistent with previously published results with *L. major* promastigotes in a different assay plate format [41],[42]. Similarly, EC_50_ values from other known leishmanicidals including paromomycin (19.7±0.6* µ*M), pentamidine (0.36±0.02* µ*M) and sodium stibogluconate (>100* µ*M) compared favorably to previously published reports with other *Leishmania* species [46],[55]. An automated, three-day variability assessment with the *L. major* promastigote drug susceptibility assay format produced Z-factors of >0.5 and >10-fold signal window. The *L. major* promastigote drug susceptibility assay was validated for automated HTS implementation by screening the 1,280 compound LOPAC set. Each compound was tested in duplicate at a single concentration (10* µ*M) and the reproducibility between the duplicate screens is represented in Figure 1 (R^2^ = 0.94). Average Z-factors were 0.71±0.03 for the two LOPAC assays, demonstrating the robustness of the developed HTS assay format. Significantly, several compounds with known *in vitro* and/or *in vivo* leishmanicidal activity were identified as primary hits, including tamoxifen, pentamidine isethionate, ketoconazole, ivermectin, niclosamide, clotrimazole, and quinacrine [1], [28], [29], [56]–[60]. We also found the leishmanicidal compounds berberine and mycophenolic acid as primary hits when we screened the UP-CMLD DP validation set [61],[62]. These data confirmed that our optimized *L. major* promastigote drug susceptibility HTS assay format could be used to identify compounds exhibiting *in vitro* as well as *in vivo* leishmanicidal activity.

The percentage of compounds in these two validation assays that were identified as growth inhibitory was relatively high, namely 10.5% and 22.6% for the diverse LOPAC and the more focused UP-CMLD DP sets, respectively, as would be expected under low stringency conditions. To test whether our screening strategy was associated with increased chemical diversity, we used the *L. major* promastigote drug susceptibility assay to interrogate the UP-CMLD diversity set, which comprised 960 compounds, at 1 and 10* µ*M. As anticipated, the total number of compounds identified as potential growth inhibitors at 10* µ*M was greater than at 1* µ*M (250 versus 46) and, importantly, 87% of the compounds identified as actives (≥50% inhibition of signal) at 1* µ*M were also found at 10* µ*M. There were more structural clusters identified at 10* µ*M (19) than at 1* µ*M (7), confirming enhanced structural diversity with the higher screening concentration. Compounds classified as singletons remained relatively consistent across the high (8) and low screening concentrations (6), although the composition of the singleton category changed with increasing screening concentration. Specifically, only 3 (of the 6) singleton compounds detected at the 1* µ*M screening concentration were represented in the 8 singletons identified at the 10* µ*M screening concentration. Thus, we adopted a high throughput, low-stringency, computationally-enhanced, small molecule screening (HILCES) strategy to maximize the structural diversity of the identified leishmanicidals.

### Interrogation of 196,146 compounds and computational enhancement of active chemotypes

**Figure 2:**
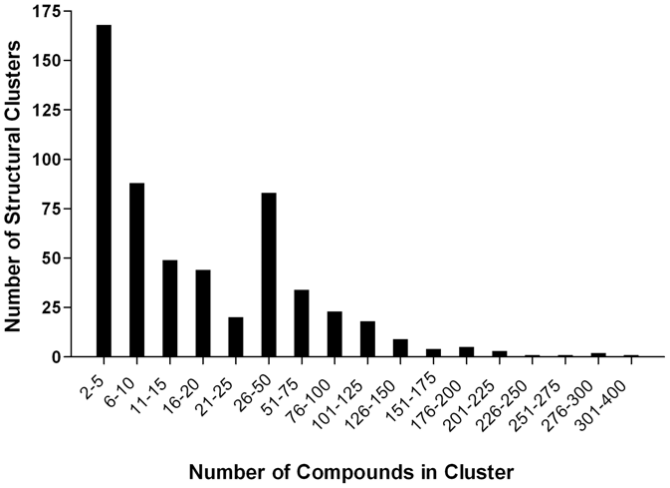
Frequency distribution of primary hit structural clusters. Active compounds identified in primary HTS activities were subjected to computational filtering by Leadscope to decrease the number of compounds entering secondary screening activities. After analyses, 553 structural clusters were identified with cluster sizing ranging from 2–360 compounds. Six hundred and forty compounds could not be assigned to a structural cluster and were classified as singletons.

We next screened 196,146 compounds at 10* µ*M in 618 plates. Performing robustly, the assay had an average Z-factor of 0.9±0.1 and an average signal to background values of 26.1±1.0 without any assay plate failures (Figure S2). Primary hits, defined as compounds that caused ≥50% inhibition of the signal readout, represented 17,629 compounds (an 8.9% hit rate). We next computationally filtered the number of compounds that would progress to secondary confirmation assays using a Jarvis-Patrick clustering methodology. We identified 553 structural clusters ranging from 2–360 members and 640 compounds as unique chemical structures (*i.e.*, singletons) (Figure 2). One compound with the smallest maximum pairwise distance to all other compounds within a cluster was selected to represent a particular structural cluster. In the 84 structural clusters consisting of two compounds, one compound was selected arbitrarily because the Jarvis-Patrick methodology is based on the similarity between several neighbors. In total, the 640 singletons and 553 representative compounds (1,193 compounds) were selected for the *L. major* promastigote secondary assays. Initially, compounds were reassayed at 10, 5, and 1* µ*M to confirm activity and assess potency quickly. One hundred forty-six compounds exhibited ≥50% inhibition when assayed at 1* µ*M and, therefore, progressed to secondary confirmation assays. All of these primary screening data have been posted for public access on the PubChem database (http://PubChem.ncbi.nlm.nih.gov/).

### Initial confirmation of growth inhibitory activity and expansion of the pool of novel leishmanicidal chemotypes

The growth inhibitory activity of the 146 compounds was confirmed using 10-point concentration (0.01–5.00* µ*M) response assays. In total, 137 compounds had EC_50_ values of <5* µ*M for an overall confirmation rate of 93.8%. Of the 137 confirmed *L. major* promastigote growth inhibitors, remarkably, 93 compounds had EC_50_ values <1* µ*M. In initial specificity studies, 70 of the submicromolar *L. major* growth inhibitors failed to inhibit the growth of the sentinel mammalian A549 cell line at 1* µ*M, suggesting specificity towards the *L. major* promastigote (Table S1). Moreover, because these compounds are part of the publicly accessible PubChem database, they have to date been screened in 99 (lowest) to 369 (highest) additional phenotypic and target-based bioassays (Table S1). Sixty-six percent of the leishmanicidal compounds registered as confirmed actives in ≤2 PubChem bioassays. None of the leishmanicidal compounds were structurally similar to the clinically used anti-leishmanial compounds sodium stibogluconate and amphotericin B (Tanimoto score ≤0.3), supporting our objective of expanding the pool of potential leishmanicidal chemotypes (Table S1). Importantly, however, compounds with previously documented *in vivo* or *in vitro* leishmanicidal activity were also identified using the HILCES system, including pentamidine isothionate, clotrimazole, aminacrine, aphidicolin, and acivicin, thus further validating our assay system (Table 1 and Table S1) [1],[42],[58],[63],[64].

**Table 1:** Effects of compounds of known pharmacological action on *L. major* promastigotes, axenic amastigote-like populations and mammalian cell lines.

Compound (Pubchem CID)	*L. major* promastigote EC_50_ (µM) (AVE±SD) Confirmation Alamar Blue	*L. major* promastigote EC_50_ (µM) (AVE±SD) Confirmation Flow Cytometry		A549 EC_50_ (µM) (AVE±SD)	HeLa EC_50_ (µM) (AVE±SD)	IMR90 EC_50_ (µM) (AVE±SD)	PC-3 EC_50_ (µM) (AVE±SD)	MDA EC_50_ (µM) (AVE±SD)	*L. major* axenic amastigote-like EC_50_ (µM) (AVE±SD) Confirmation Alamar Blue	Pharmacological Action
Acivicin (2007)	0.006±0.001		0.04±0.01	4.4±0.4	3.9±3	6.7±0.4	>50	>50	1.1±0.06	Antibiotic, antifungal, antineoplastic, antimetabolite, enzyme inhibitor
Aphidicolin (457964)	0.22±0.02		0.39±0.11	>50	>50	>50	>50	>50	0.05±0.01	Antiviral, enzyme inhibitor
Clotrimazole (2812)	0.22±0.11		0.29±0.12	>50	>50	>50	>50	>50	0.75±0.2	Local anti-infective, antifungal
Disulfiram (3117)	0.50±0.050		0.19±0.06	>50	>50	>50	>50	>50	0.13±0.01	Alcohol deterrent, enzyme inhibitor
Pentamidine Isethionate (359323)	0.29±0.05		0.73±0.39	>50	>50	>50	>50	>50	1.26±0.04	Antifungal, antiprotozoal, trypanocidal, phosphatase inhibitor
Phenytoloxamine (298107)	0.29±0.01		0.30±0.03	>50	>50	>50	>50	>50	>50	Sedating antihistamine
Sangivamycin (9549170)	0.23±0.01		0.14±0.02	0.07±0.02	>50	>50	>50	>50	4.3±0.3	Antibacterial, antibiotic, antineoplastic, kinase inhibitor
Amphotericin B (5280965) (control)	0.21±0.01		0.19±0.06	>50	>50	8.7±2.4	>50	>50	0.38±0.01	Amebicide, antibacterial, antifungal, anti-protozoal

### Characterization of leishmanicidal activity in *L. major* promastigotes and axenic amastigote-like populations

**Figure 3:**
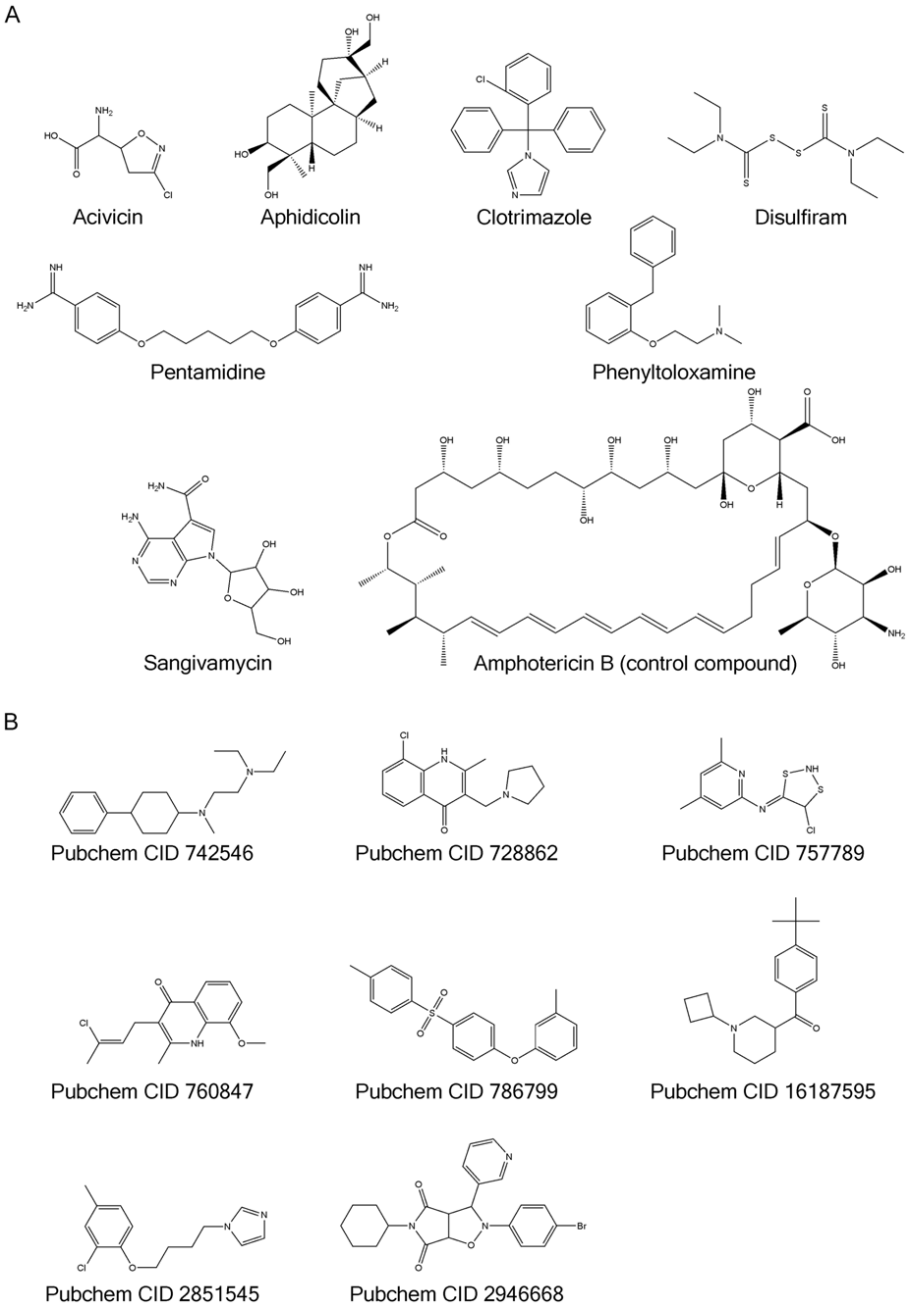
Chemical structures of test compounds. Structures of the 15 representative compounds tested empirically. Panel A, Compounds of known pharmacological action. Panel B, Compounds of unknown pharmacological action.

Next, we selected a representative group of 15 chemotypes and verified their leishmanicidal activity using compounds from a commercial supplier, thereby controlling for growth inhibitory effects resulting from any potential compound degradation during library storage. These compounds were balanced between compounds with known pharmacological actions (7) and new chemotypes (8) (Figure 3, Tables 1 and 2). We confirmed the leishmanicidal activity of the 15 chemotypes (Tables 1 and 2) with the majority of the compounds registering as submicromolar growth inhibitors. Significantly, there was a strong correlation between the EC_50_ values derived using the alamar blue assay with determinations using a flow cytometer-based format providing a second, independent methodology that confirmed the leishmanicidal activity of the test compounds (Tables 1 and 2). Subsequent testing in a human cell line panel indicated that the majority of the compounds displayed a specific and selective growth inhibitory effect toward the *L. major* parasite (Tables 1 and 2). None of the new chemotypes and only two of the compounds with known pharmacological actions, sangivamycin (PubChem CID 9549170) and acivicin (PubChem CID 2007), inhibited the growth of human cell lines tested (Table 1 and Table 2). Amphotericin B was used as a reference compound and the results were consistent with previously reported EC_50_ values (Table 1) [40],[65].

**Table 2:** Effects of compounds of unknown pharmacological action on L. major promastigotes and axenic amastigote-like populations.

Compound (Pubchem CID)	*L. major* promastigote EC_50_ (µM) (AVE±SD) Confirmation Alamar Blue	*L. major* promastigote EC_50_ (µM) (AVE±SD) Confirmation Flow cytometry	*L. major* axenic amastigote-like EC_50_ (µM) (AVE±SD) Confirmation Alamar Blue
**786799**	1.26±0.08	2.22±0.11	3.6±0.13
**742546**	0.69±0.04	0.43±0.30	>50
**760847**	0.19±0.02	0.20±0.05	0.21±0.09
**2946668**	0.86±0.16	0.35±0.06	1.2±0.4
**757789**	2.04±0.08	1.94±0.15	3.2±0.8
**2851545**	0.21±0.02	0.34±0.48	11.7±0.5
**728862**	3.63±0.96	1.77±0.12	4.3±0.8
**16187595**	0.01±0.002	0.04±0.01	2.3±0.2

We next determined the leishmanicidal activity of the 15 test compounds using an *L. major* axenic amastigote-like alamar blue-based assay. Thirteen compounds exhibited growth inhibitory activity, indicating that these compounds were active at pH 4.9. Significantly, four compounds maintained their submicromolar activity, with three compounds PubChem CID 3117 (disulfiram), 457964 (aphidicolin) and 760847, exhibiting EC_50_ values comparable to amphotericin B (Table 1 and Table 2). Several other compounds displayed EC_50_ values ≤10* µ*M.

### Additional filtering of compounds by *in silico* predictive analyses

The 15 test compounds were further classified for potential *in vivo* studies with respect to *in silico* predictive ADMET characteristics (Table S1). Twelve compounds had predicted bioavailability profiles in the good to moderate range while three compounds were predicted to have poor bioavailability. Overall, the 15 test compounds were not predicted to exhibit significant toxicity; however, two compounds (CID 786799 and 742546) have high probability for skin irritation while one compound (CID 2812) has a moderate probability of inhibiting Cyp3A4 at 10 and 50* µ*M (Table S1).

### *In vivo* leishmanicidal activity of disulfiram

**Figure 4:**
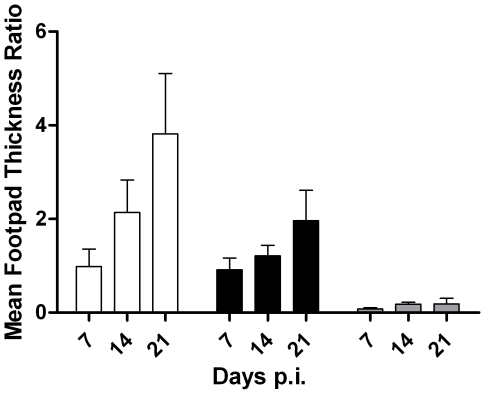
*In vivo* efficacy of disulfiram in a murine footpad model. Balb/*c* mice were infected with 10^6^ stationary phase *L. major* promastigotes (s.c.) and treated three days later with vehicle (open square), disulfiram (160 mg/kg)(black square) or amphotericin B (gray square). Footpad thickness was measured every 7 days over a 21 day period. Data are presented as mean±SEM (n = 5).

To determine if any of the new leishmanicidal chemotypes identified in the *L. major* promastigote screen had *in vivo* activity, we prioritized compounds according to the empirically-derived potency and specificity data, known pharmacological activity, activity in the *L. major* axenic amastigote-like drug susceptibility assay, *in silico* predicted ADMET, previous human usage, and novelty of the leishmanicidal chemotype. Thus, disulfiram was selected for initial *in vivo* efficacy studies. *L. major*-infected Balb/c mice were treated with vehicle, disulfiram (40 or 160 mg/kg), or amphotercin B (12.5 mg/kg) for 21 days. Drug treatment was initiated 3 days post-infection allowing for the establishment of the leishmaniasis infection. Over the course of the 10 day treatment, a decrease in the average footpad thickness was observed as compared with vehicle treated animals. With disulfiram (160 mg/kg) treatment, there was a ∼43% and 50% reduction in footpad thickness observed on days 14 and 21 post-infection, respectively (Figure 4). There was a similar decrease observed in footpad thickness with 40 mg/kg disulfiram on days 14 (25%) and 21 (35%), illustrating a dose- and time-dependent efficacy of the disulfiram treatment. As expected, the amphotericin B (12.5 mg/kg) control treatment effectively reduced footpad swelling and these data were consistent with additional experiments that showed an average 80–85% reduction in footpad swelling after amphotericin B treatment (Figure 4 and data not shown).

Although disulfiram and amphotericin B display similar levels of growth inhibitory activity in the promastigote and axenic amastigote-like assays, there was a difference in their *in vivo* effects (*i.e.*, 50% versus ∼85–90% reduction in footpad swelling). This disparity in *in vivo* effects may be the result of differences in bioavailability or mechanism of action.

## Discussion

In the current study, we illustrate the power of HILCES, a low stringency, ”forward” pharmacology, antileishmanial drug discovery strategy that employs a robust phenotypic HTS assay unencumbered by concerns for specific molecular targets [66]. HTS methodologies enabled the interrogation of a large diverse compound library, and when linked with computational methodologies, permitted refinement of the primary screening data by chemical structural clustering of chemotypes and predicted pharmacological attributes. This HILCES strategy enhanced our ability to identify novel leishmanicidal chemotypes, and, as a result, enabled us to test these new chemotypes for *in vivo* leishmanicidal activity, thus effectively expanding the pool of chemical structures that could be refined as potential leishmanicidal therapies. By capitalizing on multiple assay formats as well as *L. major* promastigote and axenic amastigote-like life cycle forms, we were able to confirm and prioritize our *L. major* growth inhibitory chemotypes for *in vivo* testing. Significantly, our preliminary studies with disulfiram indicated that our HTS and hit confirmation strategy could lead to the identification of novel leishmanicidal chemotypes with *in vivo* efficacy.

*L. major* promastigotes have frequently been used to characterize the growth inhibitory activity of potential leishmanicidal agents and they are well suited for the rapid screening of large chemical libraries due to ease of culturing [44]. In fact, two smaller scale screens, ∼2,100 compounds (http://www.sandler.ucsf.edu/lhf) and ∼15,000 compounds [67] have been performed using *Leishmania* promastigotes. Moreover, there is some evidence that the promastigote form of the parasite is an effective and reliable indicator of a compound's leishmanicidal activity in cell-based and axenic amastigotes except when examining immunomodulating anti-leishmanial compounds, such as sodium stibogluconate and meglumine antimoniate [40],[55],[68],[69]. Nonetheless, we acknowledge that there continues to be some debate about the physiological relevance of the *L. major* promastigote as an indicator of leishmanicidal activity for the cell-internalized amastigote form of the parasite, primarily because it is not the parasite stage found in humans, and they have a dissimilar response to the pentavalent antimonial compounds [40],[44]. Even so, we suggest that the promastigote-based screening assay may effectively function as the foundation for a comprehensive screening paradigm that is designed to identify and qualify novel leishmanicidal chemotypes. We recognize, however, the significance of the ability to perform HTS in a cell-based amastigote system (http://www.dndi.org/newsletters/n18/5_1.php).

The use of a publicly available annotated chemical library enabled us to cross-query a range of archived bioassays and to consider potential novel molecular targets. While the majority of the leishmanicidals failed to register as confirmed actives in other assays (Table S1), suggesting specificity for leishmanicidal activity, we found several compounds that affected previously unappreciated and provocative potential *L. major* molecular targets. For example, we found protein targets involved with cell proliferation, differentiation, invasion and motility, such as protein kinase D (gene id 5587), protein kinase C (gene id 5578), polo-like kinase 1 (gene id 5347), steroidogenic factor 1 (gene id 2516) and phosphatase regenerating liver-1 (gene id 7803) [70]–[74]. Significantly, these or related proteins are not only expressed in *L. major* but also in other parasites, including *Schistosoma mansoni* and *Trypanosoma brucei*, so they might also be critical for schistosome and trypanosome growth, differentiation, cell cycle regulation, motility and viability [31], [75]–[77]. Moreover, these data suggest that compound libraries used in conjunction with genome searches may be exploited to identify potential new drug targets.

In summary, we identified 70 submicromolar compounds that inhibit promastigote growth by using HILCES with a publicly available annotated library. Significantly, these compounds did not inhibit mammalian cell growth in companion counter-screening assays, suggesting an *L. major*-specific inhibitory response. All of the primary screening data are accessible on PubChem (http://PubChem.ncbi.nlm.nih.gov) and can be conveniently mined worldwide to allow for further refinement of individual compounds. A novel leishmanicidal chemotype, disulfiram, exhibited up to 50% *in vivo* efficacy in our animal model system. Disulfiram validated our compound screening strategy, it has a number of potential molecular targets and mechanisms. Several of the identified compounds have known molecular targets that may be relevant for this and other *Leishmania* species. The simple platform developed for *L. major* may also be useful for efforts designed to identify chemotherapeutics for other *Leishmania* species.

## Supporting Information

Figure S1 *L. major* promastigote growth curve exhibits characteristic exponential, stationary and decline phases. To develop and validate our HTS assay, we defined the growth characteristics of the *L. major* promastigote. Promastigotes were seeded at 105 parasites per mL on day 0 and the number of parasite determined for seven days. (1) Exponential growth phase; (2) Stationary growth phase; and (3) Decline. (n = 2, bars = range).
(0.18 MB TIF)

Figure S2 HTS statistics from the primary screen. Z-factors and signal to back grounds for all 618 primary screening assay plates.
(0.40 MB TIF)

Table S1 Confirmed leishmanicidal compounds as identified from the *L. major* HTS assay using the PMLSC library. ADMET - Adsorption, distribution, metabolism, excretion and toxicity; Ames - Ames test; hERG inhibition; RI - Reliability Index of in silico predicted data; SI - Skin irritation; OB - Oral bioavailability; LD50 - half maximal lethal dose; IP - intraperitoneal, OR - Oral, IV - intravenous, or SC - Subcutaneous administration; Tanimoto similarity score - method of calculating the similarity between chemical structures, SSG - sodium stibogluconate and AB - amphotericin B; AID - Assay identifier.
(0.26 MB PDF)
