# Supplementary figures and images for: Identification of Potent Chemotypes Targeting Leishmania major Using a High-Throughput, Low-Stringency, Computationally Enhanced, Small Molecule Screen
Source: PLoS Negl Trop Dis. 2009 Nov 3;3(11):e540. doi: 10.1371/journal.pntd.0000540 (PMC2765639; doi:10.1371/journal.pntd.0000540)

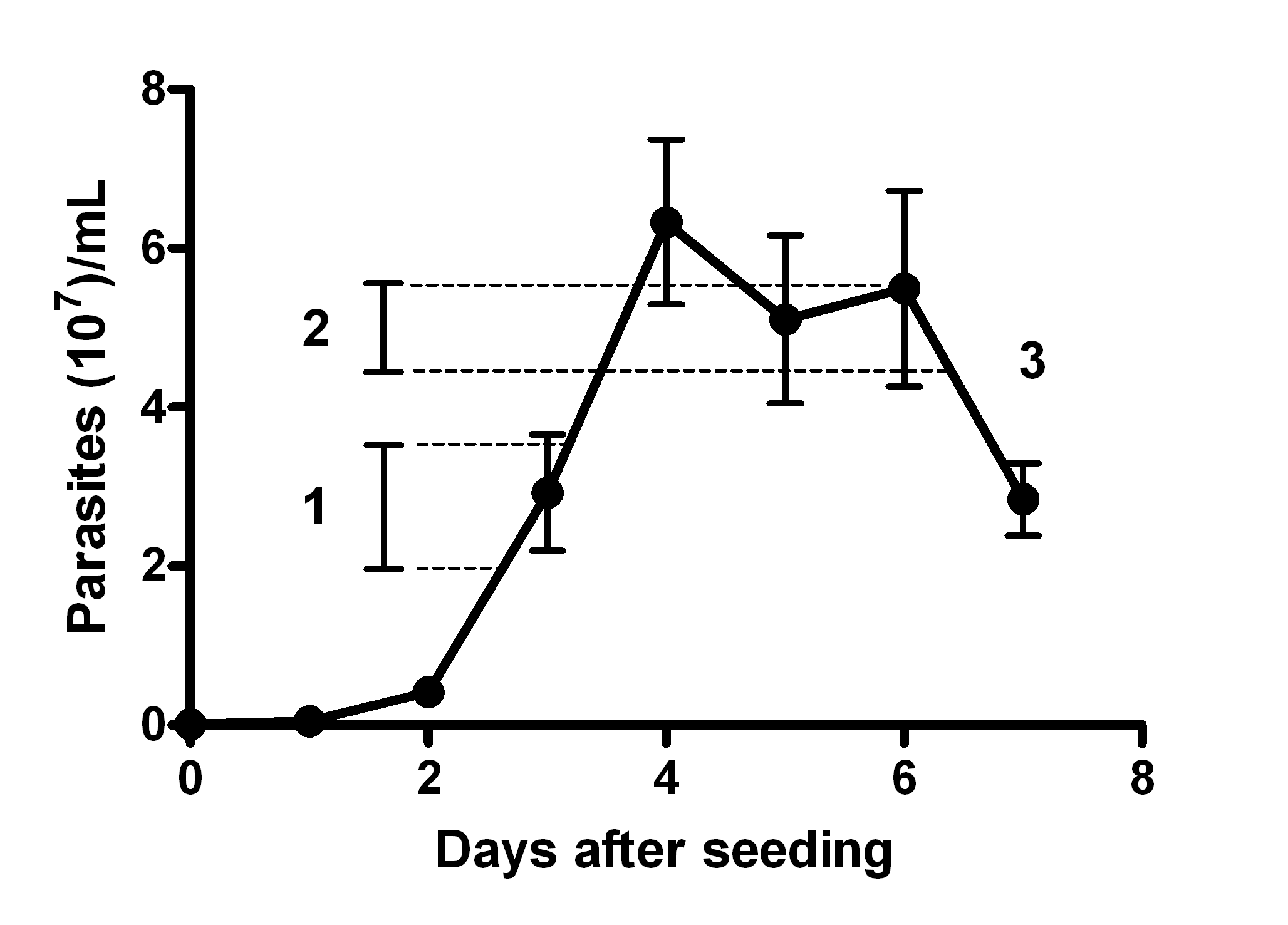

Supplement: Figure S1 — L. major promastigote growth curve exhibits characteristic exponential, stationary and decline phases. To develop and validate our HTS assay, we defined the growth characteristics of the L. major promastigote. Promastigotes were seeded at 105 parasites per mL on day 0 and the number of parasite determined for seven days. (1) Exponential growth phase; (2) Stationary growth phase; and (3) Decline. (n = 2, bars = range). (0.18 MB TIF) [file pntd.0000540.s001.tif]

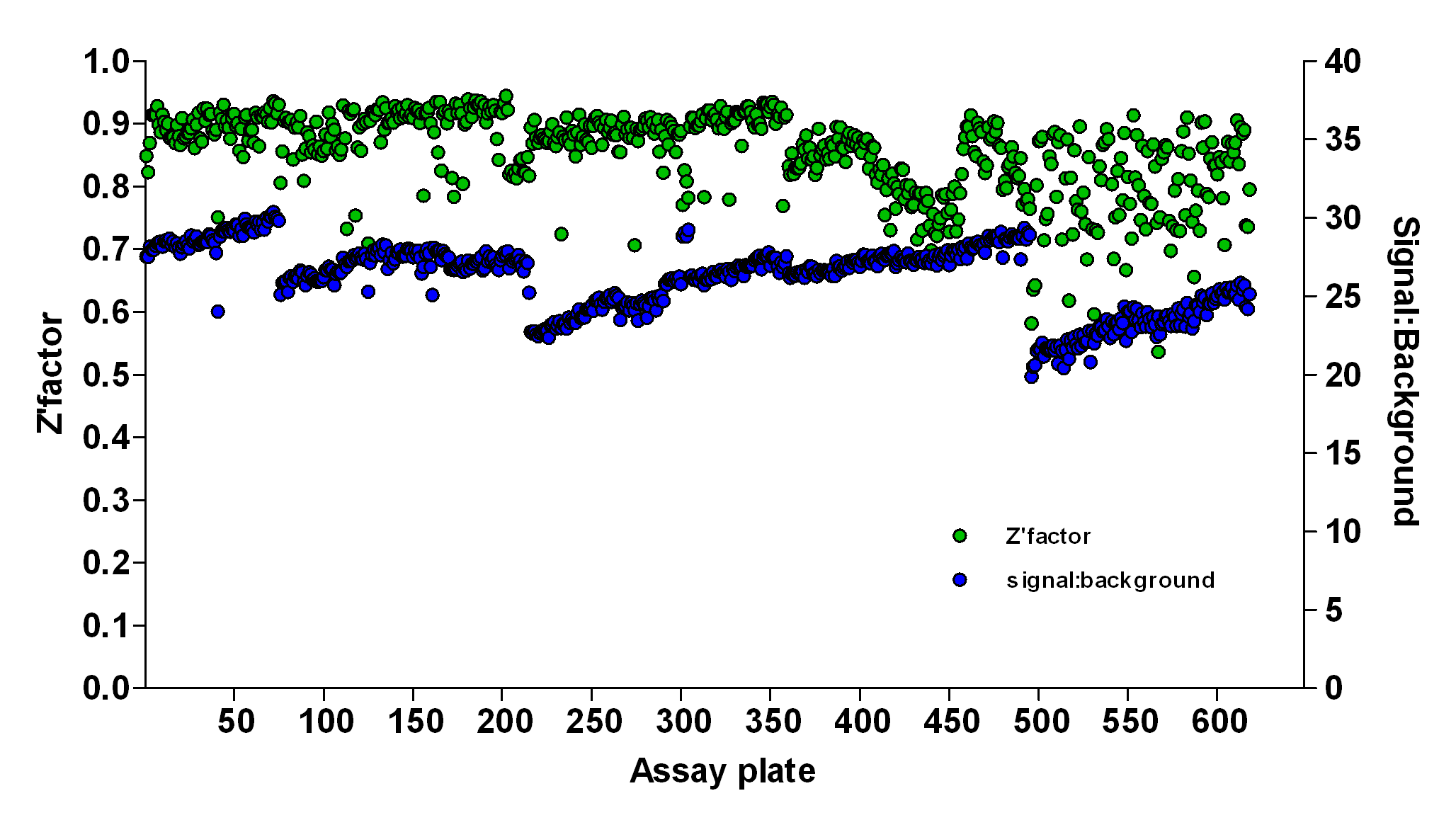

Supplement: Figure S2 — HTS statistics from the primary screen. Z-factors and signal to back grounds for all 618 primary screening assay plates. (0.40 MB TIF) [file pntd.0000540.s002.tif]
