# Supplementary material for: Identification of Potent Chemotypes Targeting Leishmania major Using a High-Throughput, Low-Stringency, Computationally Enhanced, Small Molecule Screen
Source: PLoS Negl Trop Dis. 2009 Nov 3;3(11):e540. doi: 10.1371/journal.pntd.0000540 (PMC2765639; doi:10.1371/journal.pntd.0000540)
Supplement: Table S1 — Confirmed leishmanicidal compounds as identified from the L. major HTS assay using the PMLSC library. ADMET - Adsorption, distribution, metabolism, excretion and toxicity; Ames - Ames test; hERG inhibition; RI - Reliability Index of in silico predicted data; SI - Skin irritation; OB - Oral bioavailability; LD50 - half maximal lethal dose; IP - intraperitoneal, OR - Oral, IV - intravenous, or SC - Subcutaneous administration; Tanimoto similarity score - method of calculating the similarity between chemical structures, SSG - sodium stibogluconate and AB - amphotericin B; AID - Assay identifier. (0.26 MB PDF) [file pntd.0000540.s003.pdf]

Pubchem CID	EC_{50} (nM) Ave ± SD	Pubchem AID/Active Flags/Confirmed	Predictive *in silico* ADMET: Test (RI)	$[CYP]$ IC_{50} < 50 uM, IC_{50} < 10 uM (RI)	Mouse LD_{50} (mg/kg) (RI)	Tanimoto Score (SSG/AB)
2007	Lm: 177 ± 3<br>A549: >5000	171/2/0	AMES= 0.709 (0.18)<br>hERG= 0 (0.51)<br>SI= 0.13<br>OB: Good	CYP3A4= 0 (0.79) , 0 (0.87)<br>CYP2D6= 0.02 (0.29) , 0 (0.59)	IP = 500 (0.46)<br>OR = 800 (0.15)<br>IV = 210 (0.32)<br>SC = 720 (0.43)	0.2/0.2
2812	Lm: 147 ± 30<br>A549: >5000	345/25/13	AMES= 0.207 (0.42)<br>hERG= 0.94 (0.46)<br>SI= 0.49<br>OB: Moderate	CYP3A4= 0.94 (0.63) , 0.85 (0.54)<br>CYP2D6= 0.61 (0.53) , 0.07 (0.6)	IP = 260 (0.47)<br>OR = 1000 (0.33)<br>IV = 27.74 (0.42)<br>SC = 220 (0.36)	0.03/0.02
3117	Lm: 263 ± 20<br>A549: 2163 ± 1222	160/12/3	AMES= 0.436 (0.48)<br>hERG= 0.15 (0.23)<br>SI= 0.3<br>OB: Poor	CYP3A4= 0.3 (0.56) , 0.02 (0.81)<br>CYP2D6= 0.07 (0.79) , 0.06 (0.75)	IP = 310 (0.34)<br>OR = 1700 (0.8)<br>IV = 46.74 (0.23)<br>SC = 890 (0.7)	0.04/0.02
5455	Lm: 98 ± 12<br>A549: 750 ± 2	369/89/79	AMES= 0.651 (0.47)<br>hERG= 0.04 (0.34)<br>SI= 0.32<br>OB: Moderate	CYP3A4= 0.16 (0.51) , 0.02 (0.81)<br>CYP2D6= 0.05 (0.76) , 0.04 (0.63)	IP = 150 (0.56)<br>OR = 1400 (0.81)<br>IV = 51.06 (0.42)<br>SC = 1000 (0.71)	0.04/0.02
11352	Lm: 635 ± 26<br>A549: 4699 ± 311	213/20/5	AMES= 0.968 (1)<br>hERG= 0.49 (0.23)<br>SI= 0.37<br>OB: Good	CYP3A4= 0.23 (0.33) , 0.08 (0.47)<br>CYP2D6= 0.17 (0.34) , 0.06 (0.52)	IP = 350 (0.43)<br>OR = 240 (0.33)<br>IV = 22.87 (0.58)<br>SC = 350 (0.84)	0.06/0.08
89966	Lm: 392 ± 40<br>A549: >5000	299/15/6	AMES= 0.459 (0.33)<br>hERG= 0.06 (0.28)<br>SI= 0.35<br>OB: Good	CYP3A4= 0.05 (0.77) , 0.01 (0.7)<br>CYP2D6= 0.11 (0.56) , 0.07 (0.45)	IP = 280 (0.55)<br>OR = 960 (0.49)<br>IV = 47.04 (0.41)<br>SC = 260 (0.52)	0.02/0.03
298107	Lm: 502 ± 87<br>A549: >5000	282/9/0	AMES= 0.081 (0.8)<br>hERG= 0.73 (0.48)<br>SI= 0.37<br>OB: Moderate	CYP3A4= 0.49 (0.2) , 0.06 (0.38)<br>CYP2D6= 0.84 (0.36) , 0.5 (0.08)	IP = 97.64 (0.74)<br>OR = 310 (0.56)<br>IV = 42.46 (0.88)<br>SC = 230 (0.53)	0.3/0.17
359323	Lm: 46 ± 12<br>A549: >5000	217/10/2	AMES= 0.032 (0.74)<br>hERG= 0.33 (0.47)<br>SI= 0.39<br>OB: Poor	CYP3A4= 0.48 (0.72) , 0.11 (0.84)<br>CYP2D6= 0.19 (0.59) , 0.11 (0.65)	IP = 68.71 (0.79)<br>OR = 680 (0.42)<br>IV = 28.35 (0.83)<br>SC = 92.87 (0.79)	0.2/0.1
380438	Lm: 117 ± 22<br>A549: >5000	249/3/0	AMES= 0.675 (0.23)<br>hERG= 0.21 (0.1)<br>SI= 0.99<br>OB: Moderate	CYP3A4= 0.31 (0.1) , 0.03 (0.41)<br>CYP2D6= 0.08 (0.26) , 0.01 (0.16)	IP = 360 (0.41)<br>OR = 1100 (0.48)<br>IV = 57.17 (0.33)<br>SC = 430 (0.33)	0.03/0.03
422270	Lm: 289 ± 44<br>A549: 2919 ± 320	146/10/3	AMES= 0.321 (0.11)<br>hERG= 0.45 (0.12)<br>SI= 0.74<br>OB: Good	CYP3A4= 0.35 (0.15) , 0.09 (0.18)<br>CYP2D6= 0.07 (0.33) , 0.03 (0.26)	IP = 110 (0.33)<br>OR = 720 (0.44)<br>IV = 28.74 (0.43)<br>SC = 220 (0.43)	0.06/0.05
457964	Lm: 121 ± 20<br>A549: >5000	274/18/8	AMES= 0.07 (0.43)<br>hERG= 0.02 (0.28)<br>SI= 0.28<br>OB: Good	CYP3A4= 0.04 (0.53) , 0.01 (0.71)<br>CYP2D6= 0.02 (0.58) , 0.01 (0.67)	IP = 35.64 (0.32)<br>OR = 81.07 (0.35)<br>IV = 30.33 (0.25)<br>SC = 96.64 (0.24)	0.27/0.19
460747	Lm: 202 ± 11<br>A549: 158 ± 18	224/56/19	AMES= 0.555 (0.25)<br>hERG= 0.04 (0.28)<br>SI= 0.34<br>OB: Good	CYP3A4= 0.02 (0.56) , 0 (0.61)<br>CYP2D6= 0.02 (0.55) , 0.01 (0.6)	IP = 72.05 (0.26)<br>OR = 170 (0.67)<br>IV = 20.09 (0.83)<br>SC = 46.91 (0.52)	0.08/0.07

Pubchem CID	EC_{50} (nM) Ave ± SD	Pubchem AID/Active Flags/Confirmed	Predictive *in silico* ADMET			Tanimoto Score (SSG/AB)
			Test (RI)	[CYP] IC_{50} < 50 uM , IC_{50} < 10 uM (RI)	Mouse LD_{50} (mg/kg) (RI)	
625053	Lm: 403 ± 31<br>A549: >5000	149/2/0	AMES= 0.986 (0.77)<br>hERG= 0.26 (0.09)<br>SI= 0.99<br>OB: Good	CYP3A4= 0.76 (0.27) , 0.05 (0.3)<br>CYP2D6= 0.27 (0.53) , 0.08 (0.22)	IP = 360 (0.51)<br>OR = 710 (0.51)<br>IV = 100 (0.65)<br>SC = 300 (0.49)	0.08/0.07
629413	Lm: 379 ± 77<br>A549: >5000	165/6/0	AMES= 0.246 (0.55)<br>hERG= 0.83 (0.57)<br>SI= 0.73<br>OB: Poor	CYP3A4= 0.67 (0.78) , 0.38 (0.67)<br>CYP2D6= 0.87 (0.57) , 0.34 (0.53)	IP = 59.78 (0.72)<br>OR = 230 (0.54)<br>IV = 12.79 (0.83)<br>SC = 20.46 (0.82)	0.03/0.08
644992	Lm: 255 ± 21<br>A549: >5000	178/23/8	AMES= 0.726 (0.15)<br>hERG= 0.35 (0.1)<br>SI= 0.33<br>OB: Good	CYP3A4= 0.47 (0.15) , 0.05 (0.23)<br>CYP2D6= 0.3 (0.22) , 0.17 (0.17)	IP = 350 (0.34)<br>OR = 350 (0.48)<br>IV = 49.11 (0.56)<br>SC = 73.17 (0.63)	0.06/0.07
645317	Lm: 723 ± 24<br>A549: >5000	290/3/0	AMES= 0.246 (0.16)<br>hERG= 0.24 (0.12)<br>SI= 0.32<br>OB: Moderate	CYP3A4= 0.6 (0.15) , 0.08 (0.43)<br>CYP2D6= 0.13 (0.27) , 0.05 (0.4)	IP = 320 (0.36)<br>OR = 580 (0.42)<br>IV = 71.55 (0.55)<br>SC = 180 (0.37)	0.05/0.05
657546	Lm: 845 ± 136<br>A549: >5000	321/35/15	AMES= 0.3 (0.13)<br>hERG= 0.68 (0.21)<br>SI= 0.35<br>OB: Moderate	CYP3A4= 0.49 (0.09) , 0.05 (0.3)<br>CYP2D6= 0.45 (0.29) , 0.25 (0.09)	IP = 91.92 (0.18)<br>OR = 390 (0.22)<br>IV = 22.69 (0.28)<br>SC = 140 (0.28)	0.08/0.13
728862	Lm: 392 ± 50<br>A549: >5000	274/3/0	AMES= 0.337 (0.18)<br>hERG= 0.36 (0.09)<br>SI= 0.63<br>OB: Good	CYP3A4= 0.27 (0.14) , 0.02 (0.36)<br>CYP2D6= 0.88 (0.14) , 0.34 (0.21)	IP = 390 (0.61)<br>OR = 340 (0.4)<br>IV = 79.06 (0.53)<br>SC = 270 (0.29)	0.05/0.01
742546	Lm: 827 ± 116<br>A549: >5000	162/2/0	AMES= 0.097 (0.22)<br>hERG= 0.79 (0.11)<br>SI= 0.71<br>OB: Moderate	CYP3A4= 0.17 (0.12) , 0.01 (0.44)<br>CYP2D6= 0.88 (0.17) , 0.67 (0.24)	IP = 56.8 (0.49)<br>OR = 190 (0.45)<br>IV = 79.46 (0.68)<br>SC = 340 (0.65)	0/0.02
757789	Lm: 353 ± 25<br>A549: >5000	316/20/8	AMES= 0.895 (0.35)<br>hERG= 0.27 (0.2)<br>SI= 0.32<br>OB: Good	CYP3A4= 0.2 (0.35) , 0.02 (0.38)<br>CYP2D6= 0.11 (0.22) , 0.05 (0.41)	IP = 89.1 (0.65)<br>OR = 390 (0.42)<br>IV = 16.38 (0.44)<br>SC = 58.83 (0.48)	0.08/0.05
759209	Lm: 545 ± 118<br>A549: 2576 ± 185	152/13/4	AMES= 0.738 (0.41)<br>hERG= 0.64 (0.19)<br>SI= 0.32<br>OB: Good	CYP3A4= 0.09 (0.26) , 0.02 (0.42)<br>CYP2D6= 0.08 (0.31) , 0.08 (0.33)	IP = 780 (0.36)<br>OR = 760 (0.41)<br>IV = 63.39 (0.33)<br>SC = 860 (0.48)	0.03/0.03
760847	Lm: 108 ± 23<br>A549: >5000	300/8/1	AMES= 0.432 (0.28)<br>hERG= 0.42 (0.15)<br>SI= 0.31<br>OB: Moderate	CYP3A4= 0.51 (0.17) , 0.08 (0.39)<br>CYP2D6= 0.65 (0.18) , 0.22 (0.28)	IP = 270 (0.32)<br>OR = 610 (0.27)<br>IV = 52.69 (0.28)<br>SC = 370 (0.41)	0.1/0.1
786799	Lm: 180 ± 35<br>A549: >5000	193/4/0	AMES= 0.143 (0.33)<br>hERG= 0.58 (0.22)<br>SI= 0.98<br>OB: Moderate	CYP3A4= 0.62 (0.13) , 0.21 (0.14)<br>CYP2D6= 0.08 (0.13) , 0.02 (0.28)	IP = 380 (0.45)<br>OR = 2200 (0.19)<br>IV = 82.36 (0.51)<br>SC = 250 (0.51)	0.01/0.02
871185	Lm: 287 ± 145<br>A549: >5000	314/23/7	AMES= 0.261 (0.22)<br>hERG= 0.52 (0.21)<br>SI= 0.91<br>OB: Good	CYP3A4= 0.74 (0.1) , 0.25 (0.29)<br>CYP2D6= 0.13 (0.53) , 0.02 (0.38)	IP = 800 (0.38)<br>OR = 4400 (0.35)<br>IV = 200 (0.52)<br>SC = 1800 (0.43)	0.1/0.01

Pubchem CID	EC_{50} (nM) Ave ± SD	Pubchem AID/Active Flags/Confirmed	Predictive *in silico* ADMET Test (RI)	[CYP] IC_{50} < 50 uM , IC_{50} < 10 uM (RI)	Mouse LD_{50} (mg/kg) (RI)	Tanimoto Score (SSG/AB)
952234	Lm: 205 ± 21<br>A549: 4082 ± 352	152/6/1	AMES= 0.891 (0.54)<br>hERG= 0.14 (0.23)<br>SI= 0.87<br>OB: Good	CYP3A4= 0.32 (0.18) , 0.1 (0.24)<br>CYP2D6= 0.05 (0.33) , 0.02 (0.53)	IP = 420 (0.44)<br>OR = 1700 (0.17)<br>IV = 130 (0.48)<br>SC = 510 (0.4)	0.1/0.1
1471937	Lm: 513 ± 52<br>A549: 4313 ± 540	195/13/3	AMES= 0.236 (0.06)<br>hERG= 0.9 (0.35)<br>SI= 0.29<br>OB: Moderate	CYP3A4= 0.27 (0.37) , 0.1 (0.21)<br>CYP2D6= 0.12 (0.51) , 0.16 (0.09)	IP = 690 (0.35)<br>OR = 940 (0.4)<br>IV = 87.15 (0.24)<br>SC = 210 (0.31)	0.06/0.07
1475345	Lm: 242 ± 29<br>A549: 955 ± 25	150/23/4	AMES= 0.717 (0.33)<br>hERG= 0.96 (0.16)<br>SI= 0.53<br>OB: Good	CYP3A4= 0.86 (0.27) , 0.37 (0.4)<br>CYP2D6= 0.2 (0.32) , 0.08 (0.38)	IP = 50.13 (0.42)<br>OR = 820 (0.43)<br>IV = 32.94 (0.34)<br>SC = 6600 (0.15)	0.09/0.1
1537038	Lm: 784 ± 36<br>A549: >5000	143/5/1	AMES= 0.658 (0.18)<br>hERG= 0.33 (0.11)<br>SI= 0.32<br>OB: Moderate	CYP3A4= 0.38 (0.24) , 0.09 (0.22)<br>CYP2D6= 0.35 (0.35) , 0.08 (0.31)	IP = 110 (0.6)<br>OR = 440 (0.65)<br>IV = 47.45 (0.69)<br>SC = 380 (0.5)	0.06/0.03
1736381	Lm: 87 ± 21<br>A549: 613 ± 109	99/18/6	AMES= 0.705 (0.29)<br>hERG= 0.14 (0.06)<br>SI= 0.5<br>OB: Poor	CYP3A4= 0.28 (0.29) , 0.03 (0.28)<br>CYP2D6= 0.09 (0.35) , 0.08 (0.41)	IP = 480 (0.5)<br>OR = 350 (0.08)<br>IV = 260 (0.24)<br>SC = 240 (0.36)	0.08/0.08
1811924	Lm: 166 ± 9<br>A549: >5000	170/12/2	AMES= 0.363 (0.15)<br>hERG= 0.05 (0.37)<br>SI= 0.58<br>OB: Poor	CYP3A4= 0.56 (0.38) , 0.03 (0.46)<br>CYP2D6= 0.16 (0.31) , 0.05 (0.34)	IP = 500 (0.39)<br>OR = 1100 (0.56)<br>IV = 130 (0.36)<br>SC = 340 (0.24)	0.07/0.1
1987332	Lm: 85 ± 5<br>A549: >5000	174/7/3	AMES= 0.697 (0.14)<br>hERG= 0.85 (0.23)<br>SI= 0.26<br>OB: Moderate	CYP3A4= 0.8 (0.19) , 0.08 (0.35)<br>CYP2D6= 0.04 (0.38) , 0.01 (0.39)	IP = 690 (0.63)<br>OR = 610 (0.31)<br>IV = 37.71 (0.44)<br>SC = 87.85 (0.3)	0.06/0.1
2137463	Lm: 499 ± 135<br>A549: >5000	155/7/0	AMES= 0.744 (0.13)<br>hERG= 0.19 (0.17)<br>SI= 0.31<br>OB: Good	CYP3A4= 0.26 (0.25) , 0.03 (0.36)<br>CYP2D6= 0.16 (0.33) , 0.06 (0.32)	IP = 340 (0.39)<br>OR = 470 (0.58)<br>IV = 130 (0.3)<br>SC = 410 (0.31)	0.04/0.04
2194030	Lm: 718 ± 86<br>A549: >5000	214/16/0	AMES= 0.678 (0.33)<br>hERG= 0.37 (0.08)<br>SI= 0.29<br>OB: Moderate	CYP3A4= 0.31 (0.63) , 0.1 (0.67)<br>CYP2D6= 0.1 (0.39) , 0.04 (0.5)	IP = 300 (0.59)<br>OR = 610 (0.29)<br>IV = 120 (0.61)<br>SC = 100 (0.23)	0.05/0.04
2766929	Lm: 767 ± 160<br>A549: 3546 ± 582	157/15/2	AMES= 0.165 (0.25)<br>hERG= 0.55 (0.11)<br>SI= 0.86<br>OB: Moderate	CYP3A4= 0.59 (0.24) , 0.08 (0.44)<br>CYP2D6= 0.04 (0.32) , 0.02 (0.35)	IP = 460 (0.35)<br>OR = 430 (0.21)<br>IV = 59.18 (0.24)<br>SC = 89.97 (0.23)	0.07/0.06
2846928	Lm: 35 ± 1<br>A549: 1635 ± 59	158/19/3	AMES= 0.103 (0.34)<br>hERG= 0.99 (0.51)<br>SI= 0.7<br>OB: Poor	CYP3A4= 0.09 (0.63) , 0.04 (0.75)<br>CYP2D6= 0.57 (0.47) , 0.22 (0.4)	IP = 29.59 (0.66)<br>OR = 560 (0.58)<br>IV = 27.68 (0.66)<br>SC = 440 (0.56)	0.02/0.03
2851545	Lm: 133 ± 35<br>A549: >5000	301/5/0	AMES= 0.103 (0.18)<br>hERG= 0.7 (0.34)<br>SI= 0.57<br>OB: Moderate	CYP3A4= 0.52 (0.2) , 0.45 (0.39)<br>CYP2D6= 0.67 (0.36) , 0.36 (0.19)	IP = 240 (0.44)<br>OR = 1600 (0.56)<br>IV = 61.19 (0.56)<br>SC = 440 (0.38)	0.05/0.06

Pubchem CID	EC_{50} (nM) Ave ± SD	Pubchem AID/Active Flags/Confirmed	Predictive *in silico* ADMET: Test (RI)	[CYP] IC_{50} < 50 uM , IC_{50} < 10 uM (RI)	Mouse LD_{50} (mg/kg) (RI)	Tanimoto Score (SSG/AB)
2855568	Lm: 122 ± 12<br>A549: >5000	257/5/0	AMES= 0.071 (0.35)<br>hERG= 0.19 (0.27)<br>SI= 0.85<br>OB: Poor	CYP3A4= 0.7 (0.14) , 0.11 (0.46)<br>CYP2D6= 0.1 (0.38) , 0.07 (0.34)	IP = 920 (0.64)<br>OR = 710 (0.33)<br>IV = 94.14 (0.38)<br>SC = 73.61 (0.3)	0.09/0.1
2856077	Lm: 751 ± 110<br>A549: >5000	194/9/1	AMES= 0.907 (0.58)<br>hERG= 0.45 (0.19)<br>SI= 0.91<br>OB: Moderate	CYP3A4= 0.7 (0.21) , 0.04 (0.52)<br>CYP2D6= 0.35 (0.17) , 0.17 (0.26)	IP = 630 (0.66)<br>OR = 1300 (0.54)<br>IV = 85.49 (0.74)<br>SC = 380 (0.42)	0.05/0.03
2923184	Lm: 60 ± 0.1<br>A549: >5000	163/3/0	AMES= 0.084 (0.41)<br>hERG= 0.77 (0.13)<br>SI= 0.42<br>OB: Moderate	CYP3A4= 0.25 (0.24) , 0.06 (0.41)<br>CYP2D6= 0.4 (0.27) , 0.25 (0.27)	IP = 260 (0.46)<br>OR = 440 (0.63)<br>IV = 45.62 (0.45)<br>SC = 460 (0.57)	0.1/0.1
2937831	Lm: 255 ± 48<br>A549: >5000	150/2/0	AMES= 0.331 (0.16)<br>hERG= 0.41 (0.11)<br>SI= 0.3<br>OB: Moderate	CYP3A4= 0.6 (0.21) , 0.06 (0.31)<br>CYP2D6= 0.16 (0.17) , 0.04 (0.35)	IP = 250 (0.23)<br>OR = 890 (0.31)<br>IV = 41.35 (0.43)<br>SC = 350 (0.31)	0.04/0.04
2946668	Lm: 515 ± 66<br>A549: >5000	269/5/0	AMES= 0.316 (0.36)<br>hERG= 0.1 (0.18)<br>SI= 0.27<br>OB: Moderate	CYP3A4= 0.81 (0.31) , 0.44 (0.25)<br>CYP2D6= 0.05 (0.23) , 0.01 (0.42)	IP = 470 (0.45)<br>OR = 360 (0.26)<br>IV = 71.23 (0.28)<br>SC = 260 (0.3)	0.07/0.07
2964134	Lm: 164 ± 42<br>A549: >5000	189/3/0	AMES= 0.164 (0.22)<br>hERG= 0.48 (0.15)<br>SI= 0.3<br>OB: Moderate	CYP3A4= 0.85 (0.3) , 0.38 (0.24)<br>CYP2D6= 0.83 (0.22) , 0.55 (0.21)	IP = 240 (0.57)<br>OR = 850 (0.52)<br>IV = 49.3 (0.57)<br>SC = 270 (0.27)	0.07/0.09
2999476	Lm: 187 ± 26<br>A549: >5000	304/4/0	AMES= 0.482 (0.1)<br>hERG= 0.7 (0.19)<br>SI= 0.27<br>OB: Moderate	CYP3A4= 0.94 (0.19) , 0.55 (0.23)<br>CYP2D6= 0.79 (0.32) , 0.34 (0.2)	IP = 680 (0.23)<br>OR = 570 (0.2)<br>IV = 88.86 (0.23)<br>SC = 94.7 (0.2)	0.02/0.04
3127155	Lm: 452 ± 74<br>A549: >5000	194/4/1	AMES= 0.914 (0.43)<br>hERG= 0.14 (0.29)<br>SI= 0.91<br>OB: Good	CYP3A4= 0.65 (0.16) , 0.07 (0.36)<br>CYP2D6= 0.22 (0.25) , 0.05 (0.4)	IP = 560 (0.3)<br>OR = 380 (0.47)<br>IV = 120 (0.36)<br>SC = 620 (0.41)	0.07/0.05
3164059	Lm: 218 ± 54<br>A549: 1059 ± 156	162/34/11	AMES= 0.525 (0.26)<br>hERG= 0.09 (0.23)<br>SI= 0.33<br>OB: Good	CYP3A4= 0.03 (0.46) , 0 (0.59)<br>CYP2D6= 0.02 (0.53) , 0.01 (0.59)	IP = 69.95 (0.23)<br>OR = 180 (0.66)<br>IV = 18.97 (0.8)<br>SC = 43.55 (0.52)	0.08/0.07
3236501	Lm: 105 ± 10<br>A549: >5000	332/5/0	AMES= 0.327 (0.1)<br>hERG= 0.35 (0.07)<br>SI= 0.44<br>OB: Moderate	CYP3A4= 0.71 (0.19) , 0.65 (0.19)<br>CYP2D6= 0.29 (0.13) , 0.04 (0.37)	IP = 460 (0.46)<br>OR = 1100 (0.29)<br>IV = 70.96 (0.54)<br>SC = 450 (0.38)	0.04/0.07
3237155	Lm: 743 ± 62<br>A549: >5000	246/3/0	AMES= 0.512 (0.27)<br>hERG= 0.25 (0.12)<br>SI= 0.3<br>OB: Poor	CYP3A4= 0.9 (0.39) , 0.36 (0.19)<br>CYP2D6= 0.87 (0.37) , 0.17 (0.41)	IP = 580 (0.3)<br>OR = 420 (0.16)<br>IV = 99.8 (0.38)<br>SC = 580 (0.31)	0.1/0.2
3242548	Lm: 111 ± 10<br>A549: >5000	270/2/0	AMES= 0.317 (0.1)<br>hERG= 0.82 (0.15)<br>SI= 0.87<br>OB: Moderate	CYP3A4= 0.85 (0.15) , 0.27 (0.27)<br>CYP2D6= 0.72 (0.15) , 0.37 (0.18)	IP = 110 (0.68)<br>OR = 460 (0.35)<br>IV = 76.69 (0.5)<br>SC = 200 (0.54)	0.05/0.09

Pubchem CID	EC_{50} (nM) Ave ± SD	Pubchem AID/Active Flags/Confirmed	Predictive *in silico* ADMET Test (RI)	[CYP] IC_{50} < 50 uM , IC_{50} < 10 uM (RI)	Mouse LD_{50} (mg/kg) (RI)	Tanimoto Score (SSG/AB)
3521510	Lm: 326 ± 40<br>A549: >5000	163/2/0	AMES= 0.802 (0.68)<br>hERG= 0.09 (0.19)<br>SI= 0.39<br>OB: Moderate	CYP3A4= 0.71 (0.25) , 0.14 (0.33)<br>CYP2D6= 0.14 (0.25) , 0.04 (0.33)	IP = 400 (0.26)<br>OR = 150 (0.28)<br>IV = 100 (0.57)<br>SC = 170 (0.36)	0.01/0
3536067	Lm: 636 ± 60<br>A549: >5000	163/8/2	AMES= 0.977 (0.86)<br>hERG= 0.01 (0.32)<br>SI= 0.99<br>OB: Moderate	CYP3A4= 0.22 (0.28) , 0.01 (0.47)<br>CYP2D6= 0.12 (0.28) , 0.05 (0.23)	IP = 450 (0.6)<br>OR = 640 (0.45)<br>IV = 190 (0.62)<br>SC = 520 (0.51)	0.1/0.1
3564202	Lm: 565 ± 35<br>A549: 2106 ± 77	220/27/5	AMES= 0.064 (0.55)<br>hERG= 0.94 (0.68)<br>SI= 0.29<br>OB: Moderate	CYP3A4= 0.94 (0.22) , 0.75 (0.32)<br>CYP2D6= 0.09 (0.33) , 0.02 (0.29)	IP = 270 (0.32)<br>OR = 330 (0.42)<br>IV = 56.14 (0.48)<br>SC = 72.69 (0.24)	0.03/0.03
3609942	Lm: 73 ± 13<br>A549: 2748 ± 73	205/35/12	AMES= 0.86 (0.13)<br>hERG= 0.71 (0.12)<br>SI= 0.27<br>OB: Poor	CYP3A4= 0.94 (0.09) , 0.34 (0.19)<br>CYP2D6= 0.07 (0.2) , 0.02 (0.33)	IP = 600 (0.49)<br>OR = 880 (0.55)<br>IV = 24.53 (0.52)<br>SC = 16.53 (0.17)	0.04/0.07
3769434	Lm: 504 ± 66<br>A549: >5000	152/5/2	AMES= 0.34 (0.44)<br>hERG= 0.54 (0.09)<br>SI= 0.54<br>OB: Good	CYP3A4= 0.37 (0.22) , 0.06 (0.51)<br>CYP2D6= 0.25 (0.37) , 0.08 (0.63)	IP = 310 (0.44)<br>OR = 680 (0.35)<br>IV = 91.91 (0.29)<br>SC = 190 (0.36)	0.09/0.1
3932660	Lm: 61 ± 4<br>A549: >5000	211/6/2	AMES= 0.982 (0.8)<br>hERG= 0.28 (0.08)<br>SI= 0.99<br>OB: Good	CYP3A4= 0.64 (0.17) , 0.1 (0.56)<br>CYP2D6= 0.32 (0.49) , 0.14 (0.49)	IP = 400 (0.43)<br>OR = 500 (0.69)<br>IV = 77.78 (0.3)<br>SC = 460 (0.49)	0.03/0.03
3980962	Lm: 192 ± 2<br>A549: >5000	168/5/2	AMES= 0.536 (0.22)<br>hERG= 0.49 (0.08)<br>SI= 0.9<br>OB: Moderate	CYP3A4= 0.94 (0.29) , 0.24 (0.22)<br>CYP2D6= 0.02 (0.33) , 0.01 (0.27)	IP = 980 (0.51)<br>OR = 1100 (0.45)<br>IV = 270 (0.49)<br>SC = 730 (0.38)	0.04/0.04
4202676	Lm: 767 ± 76<br>A549: >5000	152/6/2	AMES= 0.635 (0.11)<br>hERG= 0.06 (0.46)<br>SI= 0.33<br>OB: Good	CYP3A4= 0.31 (0.3) , 0.03 (0.47)<br>CYP2D6= 0.13 (0.24) , 0.05 (0.42)	IP = 430 (0.64)<br>OR = 450 (0.69)<br>IV = 210 (0.4)<br>SC = 340 (0.37)	0.06/0.04
4271960	Lm: 545 ± 193<br>A549: >5000	163/4/0	AMES= 0.525 (0.08)<br>hERG= 0.75 (0.18)<br>SI= 0.47<br>OB: Moderate	CYP3A4= 0.75 (0.13) , 0.11 (0.34)<br>CYP2D6= 0.16 (0.22) , 0.07 (0.28)	IP = 840 (0.35)<br>OR = 690 (0.31)<br>IV = 86.7 (0.49)<br>SC = 100 (0.29)	0.06/0.07
5032706	Lm: 856 ± 33<br>A549: >5000	167/2/0	AMES= 0.692 (0.24)<br>hERG= 0.17 (0.24)<br>SI= 0.31<br>OB: Good	CYP3A4= 0.27 (0.13) , 0.05 (0.36)<br>CYP2D6= 0.13 (0.13) , 0.02 (0.29)	IP = 340 (0.34)<br>OR = 250 (0.18)<br>IV = 110 (0.48)<br>SC = 390 (0.32)	0.04/0.03
5309827	Lm: 382 ± 56<br>A549: >5000	286/7/1	AMES= 0.031 (0.14)<br>hERG= 0.54 (0.09)<br>SI= 0.45<br>OB: Moderate	CYP3A4= 0.66 (0.17) , 0.03 (0.25)<br>CYP2D6= 0.08 (0.16) , 0.06 (0.24)	IP = 710 (0.39)<br>OR = 2500 (0.33)<br>IV = 83.63 (0.4)<br>SC = 550 (0.35)	0.1/0.1
5833488	Lm: 788 ± 114<br>A549: 151 ± 10	150/16/2	AMES= 0.934 (0.17)<br>hERG= 0.18 (0.07)<br>SI= 0.31<br>OB: Poor	CYP3A4= 0.7 (0.29) , 0.06 (0.36)<br>CYP2D6= 0.06 (0.28) , 0.01 (0.39)	IP = 430 (0.49)<br>OR = 720 (0.35)<br>IV = 250 (0.27)<br>SC = 80.56 (0.26)	0.1/0.1

			Predictive *in silico* ADMET			
**Pubchem CID**	**EC_{50} (nM) Ave ± SD**	**Pubchem AID/Active Flags/Confirmed**	**Test (RI)**	**[CYP] IC_{50} < 50 uM , IC_{50} < 10 uM (RI)**	**Mouse LD_{50} (mg/kg) (RI)**	**Tanimoto Score (SSG/AB)**
6261459	Lm: 306 ± 31<br>A549: >5000	170/9/1	AMES= 0.28 (0.22)<br>hERG= 0.61 (0.09)<br>SI= 0.73<br>OB: Moderate	CYP3A4= 0.44 (0.28) , 0.03 (0.52)<br>CYP2D6= 0.33 (0.14) , 0.08 (0.22)	IP = 320 (0.38)<br>OR = 380 (0.42)<br>IV = 60.82 (0.28)<br>SC = 64.07 (0.21)	0.07/0.1
6602747	Lm: 104 ± 27<br>A549: >5000	311/5/1	AMES= 0.139 (0.31)<br>hERG= 0.48 (0.12)<br>SI= 0.14<br>OB: Moderate	CYP3A4= 0.53 (0.17) , 0.06 (0.46)<br>CYP2D6= 0.7 (0.19) , 0.33 (0.1)	IP = 200 (0.4)<br>OR = 1700 (0.23)<br>IV = 43.75 (0.4)<br>SC = 840 (0.41)	0.1/0.1
6602880	Lm: 10 ± 3<br>A549: >5000	286/7/1	AMES= 0.046 (0.2)<br>hERG= 0.62 (0.5)<br>SI= 0.94<br>OB: Moderate	CYP3A4= 0.49 (0.15) , 0.1 (0.15)<br>CYP2D6= 0.44 (0.18) , 0.33 (0.18)	IP = 350 (0.65)<br>OR = 650 (0.58)<br>IV = 37.36 (0.56)<br>SC = 200 (0.43)	0.01/0.03
6603247	Lm: 83 ± 5<br>A549: >5000	305/10/2	AMES= 0.301 (0.33)<br>hERG= 0.92 (0.16)<br>SI= 0.44<br>OB: Moderate	CYP3A4= 0.69 (0.3) , 0.1 (0.12)<br>CYP2D6= 0.4 (0.24) , 0.56 (0.11)	IP = 170 (0.48)<br>OR = 660 (0.39)<br>IV = 46.76 (0.43)<br>SC = 41.63 (0.3)	0.02/0.06
6603672	Lm: 274 ± 51<br>A549: >5000	305/3/0	AMES= 0.086 (0.16)<br>hERG= 0.64 (0.25)<br>SI= 0.21<br>OB: Poor	CYP3A4= 0.88 (0.36) , 0.34 (0.26)<br>CYP2D6= 0.71 (0.28) , 0.23 (0.13)	IP = 250 (0.18)<br>OR = 970 (0.28)<br>IV = 110 (0.79)<br>SC = 330 (0.52)	0.03/0.08
6604199	Lm: 5 ± 0.1<br>A549: 1903 ± 1400	210/20/5	AMES= 0.048 (0.6)<br>hERG= 0.01 (0.31)<br>SI= 0.3<br>OB: Good	CYP3A4= 0.1 (0.49) , 0.03 (0.66)<br>CYP2D6= 0.01 (0.74) , 0.01 (0.66)	IP = 36.15 (0.62)<br>OR = 96.4 (0.4)<br>IV = 81.54 (0.78)<br>SC = 220 (0.63)	0.2/0.1
9549148	Lm: 137 ± 40<br>A549: >5000	200/7/0	AMES= 0.115 (0.27)<br>hERG= 0.88 (0.21)<br>SI= 0.39<br>OB: Moderate	CYP3A4= 0.54 (0.24) , 0.03 (0.48)<br>CYP2D6= 0.87 (0.09) , 0.63 (0.14)	IP = 150 (0.51)<br>OR = 500 (0.5)<br>IV = 27.55 (0.57)<br>SC = 270 (0.44)	0.06/0.1
9549170	Lm: 164 ± 11<br>A549: 72 ± 5	176/25/2	AMES= 0.42 (0.31)<br>hERG= 0 (0.26)<br>SI= 0.3<br>OB: Poor	CYP3A4= 0.04 (0.56) , 0.01 (0.61)<br>CYP2D6= 0.01 (0.74) , 0.01 (0.66)	IP = 220 (0.21)<br>OR = 91.34 (0.35)<br>IV = 120 (0.38)<br>SC = 720 (0.29)	0.2/0.3
9550329	Lm: 50 ± 0.1<br>A549: >5000	216/23/9	AMES= 0.112 (0.39)<br>hERG= 0.23 (0.37)<br>SI= 0.69<br>OB: Poor	CYP3A4= 0.82 (0.37) , 0.17 (0.4)<br>CYP2D6= 0.08 (0.32) , 0.02 (0.29)	IP = 120 (0.47)<br>OR = 570 (0.51)<br>IV = 32.35 (0.3)<br>SC = 220 (0.37)	0.05/0.1
11293258	Lm: 10 ± 2<br>A549: 1296 ± 76	167/23/8	AMES= 0.897 (0.58)<br>hERG= 0.96 (0.32)<br>SI= 0.39<br>OB: Moderate	CYP3A4= 0.36 (0.28) , 0.05 (0.52)<br>CYP2D6= 0.27 (0.15) , 0.08 (0.4)	IP = 160 (0.27)<br>OR = 450 (0.47)<br>IV = 37 (0.38)<br>SC = 530 (0.46)	0.03/0.04
11834429	Lm: 142 ± 32<br>A549: >5000	188/3/0	AMES= 0.095 (0.29)<br>hERG= 0.4 (0.09)<br>SI= 0.39<br>OB: Moderate	CYP3A4= 0.51 (0.16) , 0.15 (0.19)<br>CYP2D6= 0.69 (0.1) , 0.69 (0.15)	IP = 360 (0.56)<br>OR = 600 (0.54)<br>IV = 68.39 (0.53)<br>SC = 1100 (0.51)	0.06/0.07
11834470	Lm: 211 ± 47<br>A549: >5000	200/24/3	AMES= 0.641 (0.22)<br>hERG= 0.26 (0.09)<br>SI= 0.84<br>OB: Moderate	CYP3A4= 0.76 (0.12) , 0.18 (0.34)<br>CYP2D6= 0.24 (0.37) , 0.07 (0.24)	IP = 280 (0.49)<br>OR = 1100 (0.48)<br>IV = 68.33 (0.3)<br>SC = 130 (0.26)	0.1/0.09

Pubchem CID	EC_{50} (nM) Ave ± SD	Pubchem AID/Active Flags/Confirmed	Predictive *in silico* ADMET: Test (RI)	[CYP] IC_{50} < 50 uM , IC_{50} < 10 uM (RI)	Mouse LD_{50} (mg/kg) (RI)	Tanimoto Score (SSG/AB)
11948854	Lm: 607 ± 166<br>A549: >5000	180/4/0	AMES= 0.081 (0.2)<br>hERG= 0.92 (0.31)<br>SI= 0.81<br>OB: Moderate	CYP3A4= 0.39 (0.09) , 0.04 (0.21)<br>CYP2D6= 0.55 (0.3) , 0.15 (0.3)	IP = 780 (0.73)<br>OR = 270 (0.9)<br>IV = 230 (0.75)<br>SC = 210 (0.4)	0.04/0.01
11957314	Lm: 205 ± 21<br>A549: 1143 ± 53	200/26/3	AMES= 0.345 (0.1)<br>hERG= 0.98 (0.25)<br>SI= 0.71<br>OB: Poor	CYP3A4= 0.19 (0.48) , 0.06 (0.55)<br>CYP2D6= 0.75 (0.37) , 0.24 (0.14)	IP = 77.01 (0.58)<br>OR = 970 (0.51)<br>IV = 14.72 (0.68)<br>SC = 320 (0.5)	0/0.01
11958710	Lm: 174 ± 32<br>A549: >5000	193/10/1	AMES= 0.616 (0.2)<br>hERG= 0.84 (0.12)<br>SI= 0.72<br>OB: Poor	CYP3A4= 0.53 (0.14) , 0.04 (0.38)<br>CYP2D6= 0.39 (0.24) , 0.17 (0.28)	IP = 24.37 (0.38)<br>OR = 65.56 (0.69)<br>IV = 12.2 (0.22)<br>SC = 35.08 (0.3)	0.02/0.03
12004512	Lm: 746 ± 211<br>A549: >5000	162/6/2	AMES= 0.37 (0.42)<br>hERG= 0.03 (0.27)<br>SI= 0.48<br>OB: Poor	CYP3A4= 0.96 (0.86) , 0.48 (0.3)<br>CYP2D6= 0.05 (0.39) , 0 (0.41)	IP = 47.19 (0.13)<br>OR = 450 (0.42)<br>IV = 6.34 (0.23)<br>SC = 38.47 (0.33)	0.08/0.2
12004742	Lm: 491 ± 92<br>A549: >5000	181/25/10	AMES= 0.477 (0.21)<br>hERG= 0.94 (0.12)<br>SI= 0.68<br>OB: Poor	CYP3A4= 0.66 (0.17) , 0.09 (0.26)<br>CYP2D6= 0.31 (0.2) , 0.34 (0.18)	IP = 11.02 (0.06)<br>OR = 140 (0.12)<br>IV = 8.4 (0.36)<br>SC = 110 (0.25)	0.07/0.1
12005597	Lm: 629 ± 107<br>A549: 4067 ± 721	172/19/6	AMES= 0.049 (0.54)<br>hERG= 0.99 (0.56)<br>SI= 0.67<br>OB: Poor	CYP3A4= 0.09 (0.53) , 0.06 (0.66)<br>CYP2D6= 0.32 (0.38) , 0.11 (0.45)	IP = 46.51 (0.51)<br>OR = 700 (0.46)<br>IV = 23.31 (0.69)<br>SC = 350 (0.55)	0.03/0.05
13481921	Lm: 229 ± 34<br>A549: >5000	166/11/0	AMES= 0.646 (0.16)<br>hERG= 0.96 (0.19)<br>SI= 0.72<br>OB: Poor	CYP3A4= 0.4 (0.26) , 0.1 (0.58)<br>CYP2D6= 0.78 (0.5) , 0.48 (0.35)	IP = 41.73 (0.46)<br>OR = 130 (0.44)<br>IV = 18.84 (0.44)<br>SC = 29.98 (0.37)	0/0.01
15944965	Lm: 849 ± 99<br>A549: >5000	168/14/3	AMES= 0.254 (0.26)<br>hERG= 0.87 (0.13)<br>SI= 0.73<br>OB: Moderate	CYP3A4= 0.67 (0.12) , 0.11 (0.51)<br>CYP2D6= 0.69 (0.2) , 0.42 (0.17)	IP = 580 (0.39)<br>OR = 480 (0.27)<br>IV = 60.54 (0.41)<br>SC = 130 (0.32)	0.04/0.07
15944971	Lm: 505 ± 54<br>A549: >5000	166/23/5	AMES= 0.841 (0.25)<br>hERG= 0.97 (0.23)<br>SI= 0.7<br>OB: Poor	CYP3A4= 0.68 (0.12) , 0.08 (0.48)<br>CYP2D6= 0.56 (0.1) , 0.35 (0.13)	IP = 76.17 (0.15)<br>OR = 290 (0.22)<br>IV = 12.69 (0.31)<br>SC = 48.86 (0.16)	0.02/0.04
15945170	Lm: 178 ± 36<br>A549: >5000	164/2/0	AMES= 0.454 (0.19)<br>hERG= 0.22 (0.21)<br>SI= 0.16<br>OB: Poor	CYP3A4= 0.37 (0.12) , 0.01 (0.43)<br>CYP2D6= 0.28 (0.27) , 0.07 (0.28)	IP = 680 (0.48)<br>OR = 1700 (0.19)<br>IV = 13.24 (0.43)<br>SC = 1000 (0.31)	0.1/0.2
15945780	Lm: 184 ± 12<br>A549: >5000	168/10/1	AMES= 0.425 (0.14)<br>hERG= 0.8 (0.14)<br>SI= 0.3<br>OB: Moderate	CYP3A4= 0.28 (0.19) , 0.04 (0.45)<br>CYP2D6= 0.58 (0.38) , 0.26 (0.35)	IP = 24.19 (0.54)<br>OR = 550 (0.41)<br>IV = 29.82 (0.61)<br>SC = 110 (0.4)	0.03/0.03
16187569	Lm: 165 ± 25<br>A549: >5000	151/2/0	AMES= 0.188 (0.13)<br>hERG= 0.9 (0.17)<br>SI= 0.62<br>OB: Moderate	CYP3A4= 0.34 (0.16) , 0.03 (0.3)<br>CYP2D6= 0.48 (0.08) , 0.24 (0.27)	IP = 320 (0.4)<br>OR = 520 (0.58)<br>IV = 47.74 (0.41)<br>SC = 260 (0.43)	0.1/0.1

Pubchem CID	EC_{50} (nM) Ave ± SD	Pubchem AID/Active Flags/Confirmed	Predictive *in silico* ADMET Test (RI)	[CYP] IC_{50} < 50 uM , IC_{50} < 10 uM (RI)	Mouse LD_{50} (mg/kg) (RI)	Tanimoto Score (SSG/AB)
16187595	Lm: 14 ± 13<br>A549: >5000	145/2/0	AMES= 0.066 (0.29)<br>hERG= 0.34 (0.04)<br>SI= 0.7<br>OB: Moderate	CYP3A4= 0.39 (0.23) , 0.02 (0.3)<br>CYP2D6= 0.45 (0.19) , 0.24 (0.46)	IP = 180 (0.32)<br>OR = 1500 (0.57)<br>IV = 36.81 (0.45)<br>SC = 150 (0.37)	0.02/0.06
16187738	Lm: 511 ± 44<br>A549: >5000	149/2/0	AMES= 0.157 (0.2)<br>hERG= 0.68 (0.1)<br>SI= 0.48<br>OB: Moderate	CYP3A4= 0.2 (0.09) , 0.01 (0.26)<br>CYP2D6= 0.45 (0.25) , 0.33 (0.25)	IP = 300 (0.26)<br>OR = 760 (0.41)<br>IV = 36.53 (0.6)<br>SC = 340 (0.52)	0.09/0.1
16189740	Lm: 571 ± 113<br>A549: >5000	146/2/0	AMES= 0.013 (0.14)<br>hERG= 0.48 (0.07)<br>SI= 0.84<br>OB: Moderate	CYP3A4= 0.5 (0.26) , 0.1 (0.34)<br>CYP2D6= 0.29 (0.2) , 0.2 (0.27)	IP = 110 (0.7)<br>OR = 550 (0.59)<br>IV = 27.54 (0.2)<br>SC = 86.87 (0.41)	0.04/0.06
16192568	Lm: 301 ± 50<br>A549: >5000	148/2/0	AMES= 0.624 (0.5)<br>hERG= 0.14 (0.19)<br>SI= 0.3<br>OB: Moderate	CYP3A4= 0.31 (0.24) , 0.1 (0.38)<br>CYP2D6= 0.1 (0.44) , 0.01 (0.54)	IP = 1100 (0.39)<br>OR = 520 (0.59)<br>IV = 130 (0.66)<br>SC = 270 (0.69)	0.12/0.07
16192591	Lm: 357 ± 56<br>A549: 3754 ± 623	153/5/0	AMES= 0.073 (0.5)<br>hERG= 0.68 (0.14)<br>SI= 0.44<br>OB: Moderate	CYP3A4= 0.29 (0.19) , 0.04 (0.47)<br>CYP2D6= 0.34 (0.22) , 0.09 (0.26)	IP = 220 (0.6)<br>OR = 490 (0.72)<br>IV = 41.19 (0.67)<br>SC = 160 (0.37)	0.01/0.04
16196001	Lm: 50 ± 9<br>A549: >5000	146/2/0	AMES= 0.403 (0.15)<br>hERG= 0.72 (0.37)<br>SI= 0.72<br>OB: Poor	CYP3A4= 0.45 (0.39) , 0.12 (0.34)<br>CYP2D6= 0.39 (0.19) , 0.18 (0.22)	IP = 57.67 (0.55)<br>OR = 330 (0.55)<br>IV = 12.85 (0.56)<br>SC = 200 (0.33)	0.04/0.06
16196006	Lm: 703 ± 44<br>A549: >5000	145/5/1	AMES= 0.21 (0.11)<br>hERG= 1 (0.43)<br>SI= 0.64<br>OB: Poor	CYP3A4= 0.72 (0.47) , 0.32 (0.35)<br>CYP2D6= 0.79 (0.33) , 0.29 (0.34)	IP = 14.24 (0.61)<br>OR = 470 (0.33)<br>IV = 1.66 (0.41)<br>SC = 20.73 (0.36)	0.03/0.03
16196223	Lm: 18 ± 2<br>A549: 2325 ± 205	156/18/2	AMES= 0.469 (0.13)<br>hERG= 0.96 (0.4)<br>SI= 0.7<br>OB: Poor	CYP3A4= 0.78 (0.15) , 0.12 (0.2)<br>CYP2D6= 0.63 (0.33) , 0.34 (0.22)	IP = 51.41 (0.54)<br>OR = 350 (0.27)<br>IV = 11.38 (0.55)<br>SC = 74.17 (0.36)	0.01/0.05
16196319	Lm: 15 ± 1<br>A549: >5000	150/11/1	AMES= 0.304 (0.12)<br>hERG= 0.92 (0.33)<br>SI= 0.71<br>OB: Poor	CYP3A4= 0.69 (0.2) , 0.05 (0.32)<br>CYP2D6= 0.42 (0.32) , 0.29 (0.21)	IP = 23.87 (0.48)<br>OR = 400 (0.31)<br>IV = 13.55 (0.59)<br>SC = 70.87 (0.37)	0.01/0.08
